# Sister chromatid cohesion establishment during DNA replication termination

**DOI:** 10.1126/science.adf0224

**Published:** 2024-03-14

**Authors:** George Cameron, Dominika Gruszka, Sherry Xie, Çağla Kaya, Kim A Nasmyth, Madhusudhan Srinivasan, Hasan Yardimci

**Affiliations:** 1 The Francis Crick Institute; London, United Kingdom; 2 Department of Biochemistry, University of Oxford; Oxford, United Kingdom

## Abstract

Newly copied sister chromatids are tethered together by the cohesin complex, but how sister chromatid cohesion is coordinated with DNA replication is poorly understood. Prevailing models suggest cohesin bound to DNA before replication establishes cohesion by replisome passage through the cohesin ring or by transfer of cohesin behind the replication fork by replisome components. By visualizing single replication forks colliding with pre-loaded cohesin complexes, we find that cohesin is instead pushed by the replisome to where a converging replisome is met. Whilst the converging replisomes are removed during DNA replication termination, cohesin remains on nascent DNA. We demonstrate that these cohesin molecules tether the newly replicated sister DNAs together. Our results support a new model where sister chromatid cohesion is established during DNA replication termination.

The cohesin complex tethers sister chromatids together from the moment they are generated in S phase until their separation in anaphase. This fundamental phenomenon, called sister chromatid cohesion, underpins orderly chromosome segregation. Cohesin is a ring-shaped complex, with four core subunits (SMC1, SMC3, RAD21^Scc1^ and SA1/SA2^Scc3^) (1). In addition to the essential role of cohesin in sister chromatid cohesion, cohesin organizes interphase chromosomes by loop extrusion (2, 3). Cohesin is loaded onto chromatin by the NIPBL/MAU2 (Scc2/4) loader (4), which in vertebrates interacts with pre-replication complexes (pre-RCs) (5, 6). In eukaryotes, origins of replication are licensed during G1 phase through the formation of pre-RCs, which contain inactive double hexamers of MCM2-7 (7). In S phase, pre-RCs are remodeled to form CDC45-MCM2-7-GINS (CMG) helicases, which then unwind DNA. The replisome complex, containing further components, is assembled around the CMG helicase (8, 9). Cohesion is thought to arise from co-entrapment of sister DNAs within cohesion rings (1, 10). Cohesion establishment is strictly limited to S-phase (11), and is believed to be mechanistically coupled to DNA replication, involving two independent pathways (12, 13). A ‘conversion’ pathway uses pre-loaded cohesin complexes associated with parental DNA ahead of the replication forks to form cohesive structures, while a ‘*de novo*’ pathway uses cohesin rings loaded onto DNAs during S phase. The molecular mechanisms by which cohesion is generated by these two pathways are unclear, which constitutes a major gap in our understanding of eukaryotic biology.

Two types of mechanism have been envisaged for conversion. Cohesion could be generated by passage of the replisome through cohesin rings that had previously entrapped unreplicated DNA (fig. S1A, left-hand side). Alternatively, cohesin rings could be transferred from unreplicated to replicated DNAs while remaining associated with the replisome (fig. S1A, right-hand side). Both these scenarios predict that DNA-associated cohesin rings, upon encounter with the replisome, would remain behind advancing replication forks. Testing this prediction *in vivo* has been challenging because cohesin is constantly mobile on DNA (14), new cohesin is loaded onto DNA during most of the cell cycle (15), and cohesin-replisome encounters are stochastic due to the nature of origin firing in eukaryotes (7). To determine the fate of pre-loaded cohesin during DNA replication, we performed live visualization of single replication forks encountering cohesin complexes.

## Results

### Pre-loaded cohesins are pushed by replisomes

**Fig. 1:**
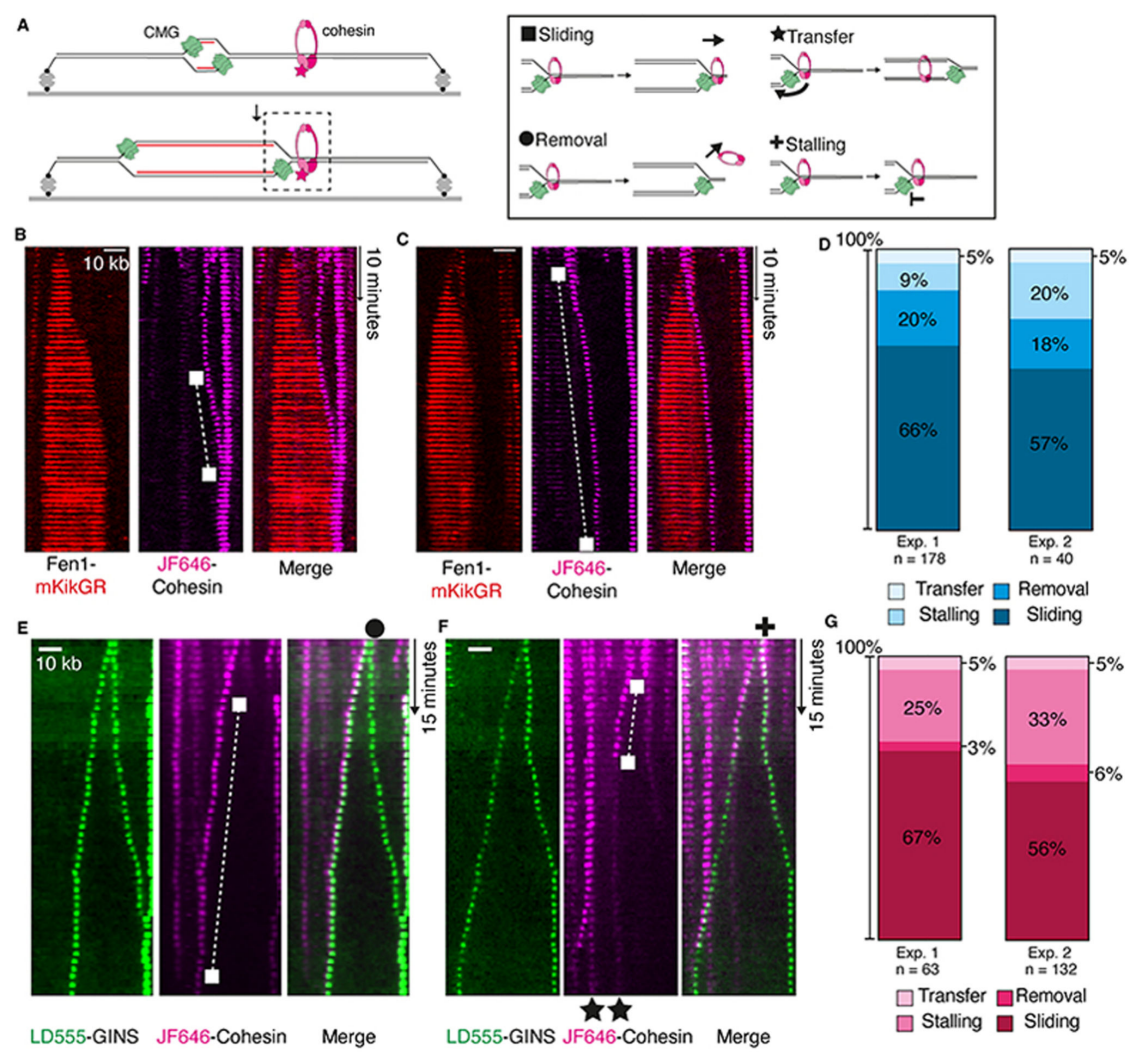
Replisomes push cohesin during DNA replication. (**A**) Cartoon showing DNA replication from a single origin on surface-tethered λ DNA. Replication is performed in *Xenopus laevis* egg extracts while collisions between replisomes and cohesins are visualized. (**B** and **C**) Collisions between replication forks, labelled with Fen1-mKiKGR (red), and pre-loaded JF646-cohesin (magenta) are visualized. Examples showing cohesin sliding ahead of a Fen1-mKikGR-labelled replication fork. (**D**) Comparison of primary cohesin fate after collision with replication forks in extracts. Two independent experiments are shown. (**E** and **F**) Representative kymograms showing LD555-GINS collision with JF646-cohesin. Examples of different cohesin fates (sliding, removal, transfer and fork stalling) are marked with symbols. (**G**) Proportions of cohesin fates after collision by labelled replisomes. Two independent experiments are shown.

To study the outcome of replisome collision with cohesin in a physiologically relevant setting, we used *Xenopus laevis* egg extracts (16–18), which contain all factors needed for *in vitro* DNA replication and repair (19) whilst supporting cohesion establishment (20). Two different extracts allow a single round of DNA replication to be performed. High-speed supernatant (HSS) is used to license DNA with pre-RCs in a sequence-independent manner, while nucleoplasmic extract (NPE) is used to replicate DNA. We used an assay developed to visualize fluorescent molecules during replication of surface-immobilized DNA in egg extracts (17, 18, 21) to assess the outcomes of replication fork encounters with cohesin (Fig. 1A and fig. S1B). Fluorescently labeled recombinant *Xenopus laevis* cohesin (fig. S1C) loaded onto chromatin in extracts in a manner dependent on DNA being licensed with pre-RCs and at comparable levels to endogenous cohesin (5, 6) (fig. S1D). Cohesin labeled with Janelia Fluor 646 (JF646-cohesin) was loaded onto surface-tethered λ DNAs. Replication fork progression was followed in extracts by observing nascent DNA with fluorescently tagged Fen1 (Fen1-mKikGR). To monitor encounter of individual replication forks with pre-loaded cohesin, origin firing was restricted using p27^kip^, a CDK inhibitor (fig. S1B).

Prevailing models for cohesin conversion predict incorporation of pre-loaded cohesin into nascent DNA behind the replication fork. Unexpectedly, in our conditions, this outcome of cohesin transfer occurred only in 5% events (Fig 1, B to D, Movie S1, and fig. S2, A to C). In most cases, we instead observed that pre-loaded cohesin was pushed ahead of forks (cohesin sliding, 57-66% of events). In 18-20% of events, cohesin was removed shortly after encounter with the replication fork and in 9-20% of events replication fork stalling was detected upon encounter with pre-loaded cohesin. The lack of cohesin transfer was surprising as extracts contain all factors needed for cohesion establishment by the conversion pathway. We also observe a notable dissociation of cohesin in extracts in a replication-independent manner, likely due to competition for DNA binding between cohesin and the many other DNA-binding factors in extracts and the presence of cohesin unloader (fig. S3).

We next investigated if endogenous cohesin in *Xenopus* egg extracts interferes with the transfer of DNA-loaded fluorescently-tagged cohesin during replication. Previously we have shown that parental histone transfer behind replication forks is reduced by soluble histones present in extracts, which likely inhibit parental histone interaction with replisome components (22). To test if endogenous cohesin in extracts could have a similar inhibitory effect on cohesin transfer, it was immunodepleted from extracts, which had a negligible effect on fork speeds (fig. S4, A and B). Importantly, even in cohesin-depleted extracts, cohesin transfer was rare and 58-68% of cohesin was pushed by forks (fig. S4, C to F). This suggests that endogenous cohesin complexes in extracts do not prevent transfer of pre-loaded cohesin.

We also considered if the presence of high concentrations of Fen1-mKikGR, which result in PCNA being retained on DNA during replication (17), inhibited cohesin transfer. Because PCNA has been implicated in cohesion establishment (23), we omitted Fen1-mKikGR to exclude the possibility of inefficient cohesin transfer due to improper PCNA retention. We visualized the replisome directly using a method previously described (24). Purified fluorescent GINS was used to rescue DNA replication in GINS-depleted extracts (fig. S5). During replication of λ DNA from single origins, fluorescent CMG moved at the tip of Fen1-mKikGR tracts (24–26), and at an average speed consistent with previous work (426 bp/minute; fig. S6, A to C). We next visualized the outcomes of collisions between fluorescent replisomes and pre-loaded JF646-cohesin (Fig. 1, E and F, Movie S2, and fig. S6, E to G). Under these conditions, cohesin transfer was still very rare (5% of events; Fig. 1G) and cohesin sliding ahead of the replisome dominated (56-67% of events). Therefore, the low frequency of cohesin transfer observed in our previous experiments was not caused by Fen1-mKikGR.

### Cohesins relocalize to DNA replication termination sites

**Fig. 2:**
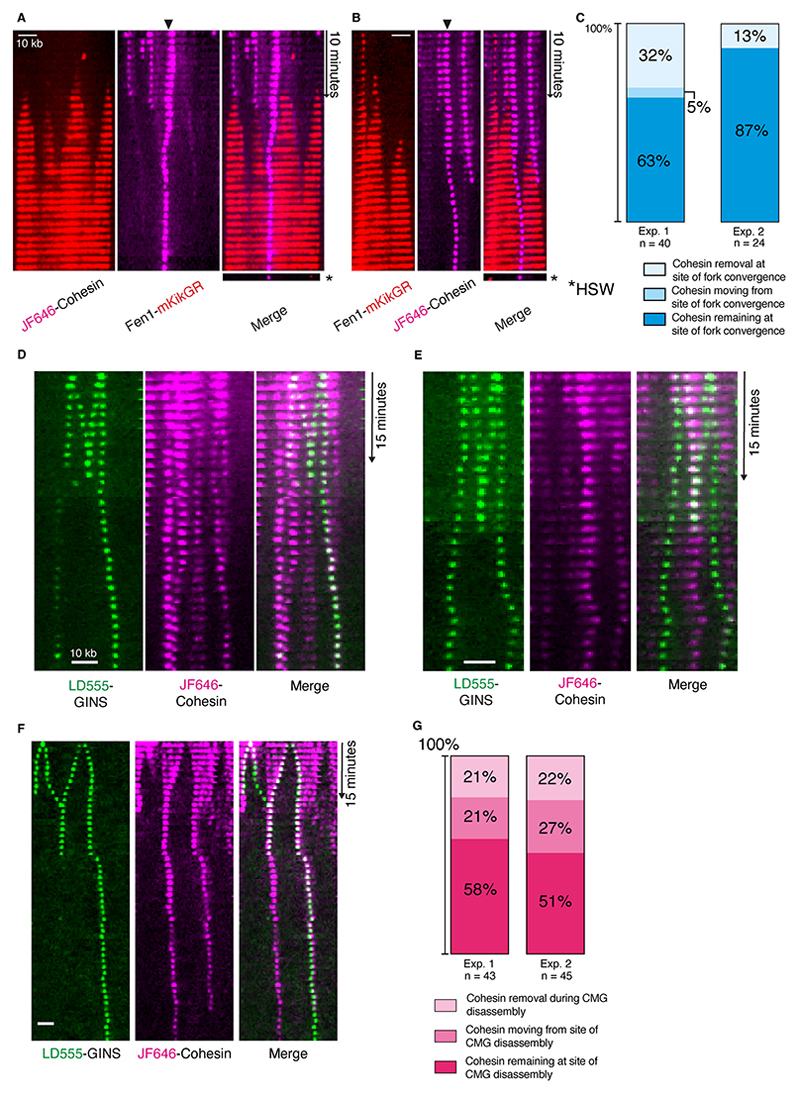
Cohesin is pushed to positions of DNA replication termination. (**A** and **B**) Kymograms showing replication forks colliding with JF646-cohesin complexes under conditions of high origin firing. After a period of replication, a high salt wash (HSW) was performed and the same DNAs were imaged. (**C**) Quantification of cohesin fates at converging replication forks. The fate of cohesin that was pushed to a converging replication fork was measured. Two independent experiments are shown. (**D** to **F**) Kymogram examples showing replisome (LD555-GINS) progression on DNA from multiple origins and colliding with JF646-cohesin. (**G**) Quantification of JF646-cohesin fate at sites where converging replisomes (LD555-GINS) are removed, with two independent experiments shown.

If pre-loaded cohesin is not transferred behind the replisome onto the replicated sister DNAs, how does conversion generate cohesion? We speculated that cohesin pushed ahead of the replisome could generate cohesion when meeting a converging replisome. To determine the fate of cohesin during fork convergence, replication was started from multiple origins (fig. S7A). When visualizing converging replication forks using Fen1-mKikGR, the majority of cohesin pushed ahead of replication forks remained in positions where converging replication forks met (Fig. 2, A to C, Movie S3, and fig. S7). In some cases, fork convergence was accompanied by either cohesin eviction or further cohesin movement after fork convergence (Fig. 2C). Cohesin remaining on DNA after fork convergence was resistant to a high-salt wash that removed Fen1 from DNAs (Fig. 2, A and B, and fig. S7, B and C), suggesting that this population of cohesin was topologically bound to DNA (27). On fully replicated DNAs, we observed multistep photobleaching of cohesin in diffraction-limited spots (mean = 1.90 steps, fig. S8, A to F), suggesting multiple cohesins are pushed to sites of replication fork convergence. We envisaged that upon fork convergence, replisomes disassemble while cohesin traps both daughter strands together. To test this, multiple origin firing experiments were performed with fluorescent CMG. As expected, replisomes were disassembled shortly after fork convergence (26) (fig. S9A), even when labeled cohesin persisted on DNA (Fig. 2, D to F, Movie S4, and fig. S9, B to E). Strikingly, in 51-58% of cases, cohesin remained at the site of replisome disassembly (Fig. 2G). Cohesin signal lifetime on DNA after replication termination varied, on average remaining for 43.7 min (±17.8 min) (fig. S8, G to I). We conclude that cohesin complexes are pushed by advancing replisomes to sites of fork convergence and remain at these sites after replisome disassembly. The key question is therefore: do the cohesin rings that persist on DNA after replication termination in our assay mediate cohesion?

### Cohesin complexes retained at replication termination sites can tether sister DNAs

**Fig. 3:**
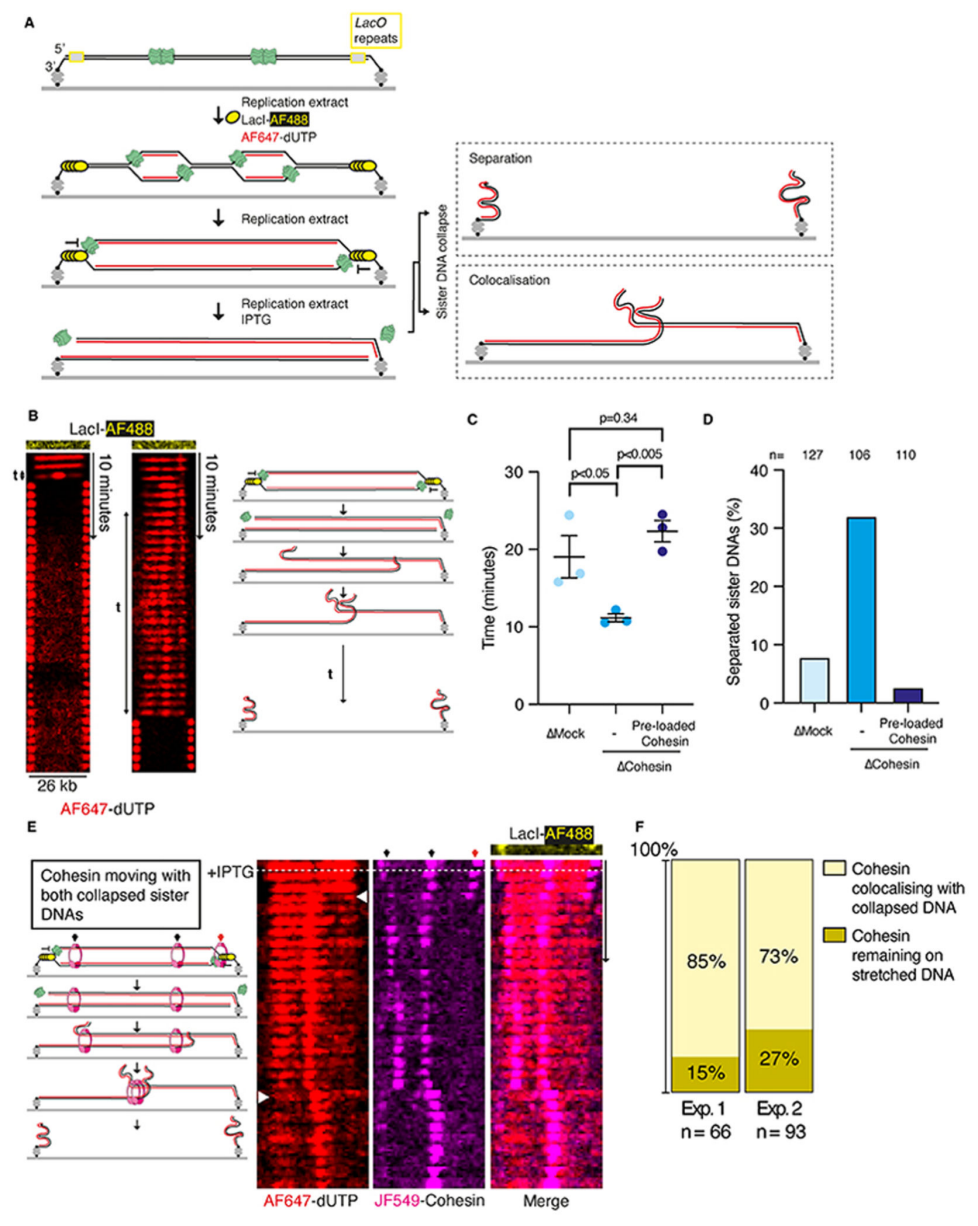
Cohesin holds together newly replicated surface-tethered sister DNAs. (**A**) Diagram showing sister DNA collapse experiments. Replication forks are paused at DNA ends by LacI. Upon IPTG addition replication forks reach DNA ends and collapsing sister DNAs are visualized. (**B**) Example kymograms where both sister DNA strands collapse (see cartoon). The time that collapsed DNA strands colocalized before separating is indicated. (**C**) Time that collapsed DNA strands colocalized under different extract depletion conditions. n=3 independent experiments were performed, with the following number of individual molecules: Mock (n=32, 36, 59), Depletion (n=13, 40, 53), and Rescue (n=16, 41, 53). Data are mean ± SEM, compared with a two-sided *t-*test. (**D**) Percentage of sister DNAs that have separated from one another within 2 minutes of collapsing from both ends under different extract depletion conditions. (**E**) Example kymogram showing JF549-cohesin associating with both collapsed sister DNAs. The red arrow indicates cohesin at the end of DNA tethers, which is excluded from the analysis. (**F**) Quantification of cohesin position after sister DNA collapse of either one strand or both strands, with data from two independent experiments shown.

To assess if cohesin molecules at replication termination sites provide cohesion, we developed an assay to measure sister DNA cohesion. Previous experiments tethering linear DNAs to the surface only via 3’-biotins have shown the new sister DNA that does not contain biotin collapses from the surface after the replisome reaches the 5’-end (fig. S10A) (24). Using this knowledge, we designed a DNA template to visualize interaction between sister DNAs after replication (Fig. 3A). Binding of Alexa Fluor 488 labelled LacI (LacI-AF488, fig. S10B) to 48 *lacO* repeats at each DNA end (fig. S10C) blocks replisome progression (Fig. 3A). Subsequent removal of LacI-AF488 by IPTG addition results in synchronous completion of replication and collapse of both sister DNAs (Fig. 3A). Importantly, this set up enables us to measure cohesion between the replicated sister DNAs. Lack of cohesion between the sister DNAs would result in sister DNAs immediately separating (Fig. 3A, right panel, top scenario). If, however the replicated DNAs were held together, the two collapsed sister DNAs would colocalize (Fig. 3A, right panel, bottom scenario).

We initiated replication from multiple origins in the presence of LacI-AF488, and Alexa Fluor 647-dUTP (AF647-dUTP) was incorporated into nascent DNA for visualization. Excess LacI-AF488 and AF647-dUTP were washed away and replication extract containing IPTG was added to release LacI-AF488 from DNA ends. Under these conditions, regions of AF647-labelled replicated DNA could be visualized during synchronous collapse of new sister DNAs from DNA ends. The assay is intrinsically validated by molecules with partially replicated DNA, where the labeled nascent DNA approaches only one DNA end. In these instances, after removal of LacI, the collapsed sister DNA moved with the replisome towards the opposite end of the DNA (fig. S10D), as observed previously (24). Crucially, on molecules where the DNA template was fully replicated up to the LacI barrier at both ends, LacI removal caused collapse of sister DNAs from both ends (fig. S10E). When both new sister DNAs collapsed, they colocalized together for varying length of time before separating, giving a measure of cohesion. The critical question is whether colocalization of sister DNAs resulted from cohesin-mediated cohesion.

To test whether cohesin complexes physically tether collapsing sister DNAs in the experiments described above, the assay was performed in either mock- or cohesin-depleted extracts (fig. S11A), and we compared the time that collapsed sister DNAs colocalized (Fig. 3, B to D, Movie S5, and fig. S11, B and C). The collapsed sister DNAs remained associated significantly longer in mock-depleted extracts compared to cohesin-depleted extracts (Fig. 3C). Importantly, when purified cohesin was pre-loaded onto DNAs before replication in cohesin-depleted extracts, collapsed sister DNAs remained together for periods of time comparable to that in mock-depleted extracts. After collapse, 32% of sister DNAs were separated within 2 minutes in cohesin-depleted extracts versus 3% when cohesin was pre-loaded before replication in depleted extracts (Fig. 3D). In a bulk replication assay, formation of replicated supercoiled plasmid was indistinguishable between mock- and cohesin-depleted extracts (fig. S12, A to C), eliminating the possibility that tethering between sister DNAs may be due to cohesin delaying replication completion at converging forks. Furthermore, the majority of colocalizing sister DNAs were disrupted when treated with 0.1% SDS (fig. S12, E to F) indicating that sister DNAs are coupled through DNA-protein interactions rather than DNA-DNA interactions. These results show that cohesin complexes pre-loaded onto parental DNA physically tether sister DNAs after replication. Taken together with our previous observation that pre-loaded cohesin is predominantly pushed to sites of replication termination and remains at these sites after replisome disassembly, our data provide strong evidence that cohesion establishment by cohesin conversion occurs during replication termination.

Our model also predicts that cohesive cohesin complexes should be dragged by the collapsing sister DNAs and remain associated with both sister DNAs (fig. S13A, right panel, top scenario). If cohesin does not tether sister DNAs together, we would expect half of the cohesin molecules to remain associated with stretched DNA after sister DNA collapse (fig. S13A, right panel, bottom scenario). To test this, sister DNA collapse experiments were performed with JF549-cohesin pre-loaded onto parental DNA and imaged simultaneously with AF647-dUTP. Cohesin was observed to move when both sister DNAs collapsed (Fig. 3E, Movie S6, and fig. S13B) and when a single sister DNA collapsed (fig. S13C). Strikingly, 73-85% of cohesin molecules moved and colocalized with collapsed sister DNAs (Fig. 3F), while only 15-27% remained on stretched DNAs (fig. S13D). We estimate that 50-70% of cohesins retained on DNA after replication termination bound both sister DNAs (see Methods). The cohesin colocalization with collapsed sister DNAs reaffirms that cohesion establishment by pre-loaded cohesin complexes happens during replication termination.

### Replisome disassembly during replication termination is not critical for cohesion

**Fig. 4:**
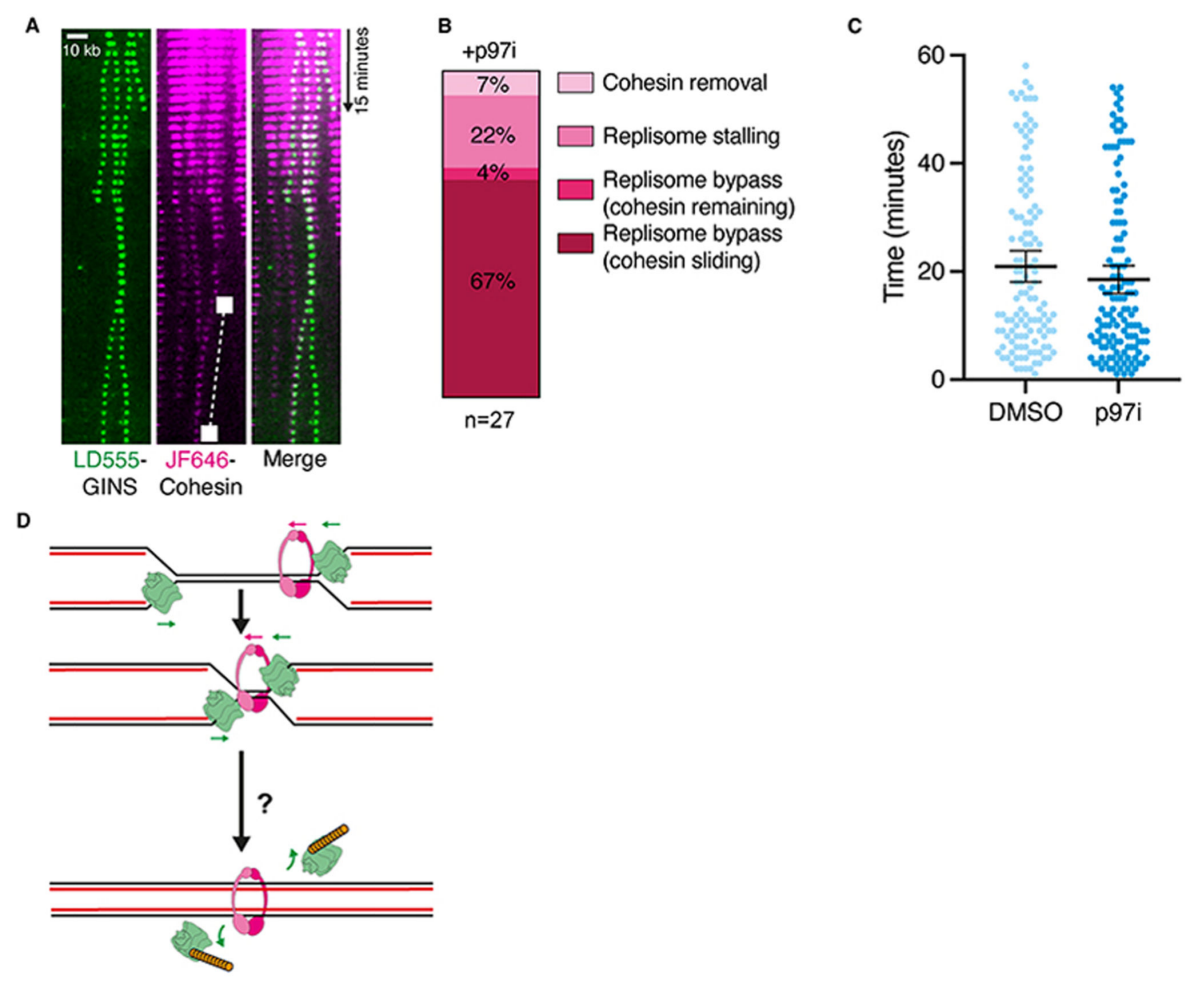
Cohesin dynamics during replication termination when replisome disassembly is inhibited. (**A**) Replisomes were labeled directly using LD555-GINS and replisome disassembly upon fork convergence was inhibited with 200 μM p97i (NMS-873). In this example, converging replisomes bypass one another and one replisome continues pushing a labelled cohesin (white square). (**B**) Quantification of cohesin and replisome fates observed during replication termination when replisome disassembly is inhibited. (**C**) Plots showing timings of sister chromatid colocalization ±p97i. Data were collected from two experiments for each condition (DMSO, n = 124 and p97i, n = 140). (**D**) Model of cohesion establishment during replication termination. Possible scenarios describing termination-coupled cohesion establishment are shown in fig. S15.

A number of replisome components including TIMELESS/TIPIN (Tof1/Csm3) and AND-1 (Ctf4) have roles in both cohesion establishment and replisome disassembly (13, 28). Therefore, we considered the possibility that timely replisome disassembly could be required for proper cohesion establishment. To test this idea, we visualized replisomes and cohesin complexes during fork convergence while blocking replisome disassembly with an inhibitor of p97 (p97i) (29). After fork convergence, replisomes stably bound DNA and in some cases bypassed one another (26). Where cohesin was pushed ahead of one or both replisomes before fork convergence, the majority of replisomes bypassed one another with cohesin co-localizing with one of the replisomes (Fig. 4, A and B, Movie S7, and fig. S14, A and B). In some cases, replisomes stalled and did not bypass one-another with cohesin remaining (fig. S14C). Cohesin removal during replisome convergence (fig. S14D) and cohesin remaining between bypassing replisomes (fig. S14E) were rarely observed. As cohesin was generally stable on DNA when replisome unloading was inhibited, we assessed whether cohesin provided cohesion in the presence of p97i. Sister DNA collapse assays were repeated (fig. S14, F and G), and comparing the time collapsed sister DNAs colocalized showed no significant difference when adding p97i (Fig. 4C). These results suggest that when replisome disassembly is inhibited in our assay, remaining cohesins can still provide cohesion between sister DNAs. Therefore, the role of the replisome components TIMELESS/TIPIN (Tof1/Csm3) and AND-1 (Ctf4) in replisome disassembly is distinct from their role in cohesion. Interestingly, abrogation of the conversion pathway in yeast leads to eviction of DNA-associated cohesin rings during replication (13). We therefore speculate that cohesion establishment factors such as TIMELESS/TIPIN may be needed to facilitate cohesin sliding to replication termination sites perhaps by interacting with the cohesin complex (30).

## Discussion

In contrast to prevailing models, which place pre-loaded cohesin behind replication forks, we found that cohesin rings are pushed along the DNA by the advancing replisome. Cohesin rings pushed to positions of fork convergence are retained on replicated DNA even after replisome disassembly and are capable of tethering sister DNAs together. The notion of replisomes pushing cohesin is supported by transcription repositioning cohesin on yeast chromosomes (14) and cohesin being pushed ahead of T7 RNAP and FtsK *in vitro* (31, 32). A previous single-molecule study reported that a significant fraction of cohesin was incorporated into replicated DNA in *Xenopus* egg extracts (33). We suggest that the cohesin transfer events they observed were largely the result of cohesin being incorporated during replication termination, as the study used firing from multiple origins. In our system, conversion of pre-loaded cohesin to cohesive structures via *bona fide* cohesin transfer behind the replication fork might occur, albeit rarely. Removal and stalling events might reflect differences in cohesin binding onto DNA before replication. We envisage that cohesin removal helps prevent accumulation of many cohesins sliding ahead of the fork, which could impede replisome interaction with other DNA-bound proteins such as histones.

Our data strongly support a mechanism where cohesion establishment using pre-loaded cohesin rings occurs at the sites of replication termination and is independent of CMG unloading. We propose several potential models for cohesion establishment at termination sites (Fig. 4D). Converging replisomes could pull the final stretches of unreplicated DNA through cohesin rings (fig. S15A). This would result in replisome disassembly and cohesion between new sister DNAs. This model negates the requirement for replisomes to pass through cohesin rings or for transient ring opening. Alternative possibilities are that cohesin is transferred behind one replication fork during termination (fig. S15B), or a terminating replisome bypasses the cohesin ring (fig. S15C). Bypass of the cohesin ring could use a mechanism similar to CMG bypass of DNA-protein crosslinks (24). Factors required for cohesin conversion (13) could aid pushing of cohesin by the replisome, or be involved in the molecular transactions between DNA, replisomes and cohesin during replication termination. Further work will be required to dissect the exact nature of cohesion establishment during DNA replication termination.

## Supplementary Material

fig. S1

Movie S1

Movie S2

Movie S3

Movie S4

Movie S5

Movie S6

Movie S7

Supplementary Movies

## Data Availability

All data needed to evaluate the conclusions in the paper are present in the paper and/or the Supplementary Materials. Additional data related to this paper may be requested from the authors.

## One-Sentence Summary

Cohesins are pushed by replication forks and establish cohesion at DNA replication termination sites.
