# Supplementary material for: Sister chromatid cohesion establishment during DNA replication termination
Source: Science. Author manuscript; Available in PMC 2024 Apr 5. (PMC7615807; doi:10.1126/science.adf0224)
Supplement: fig. S1 [file EMS194777-supplement-fig__S1.pdf]

Science
AAAS

Supplementary Materials for

Sister chromatid cohesion establishment during DNA replication termination

George Cameron *et al.*

Corresponding authors: Madhusudhan Srinivasan, madhusudhan.srinivasan@bioch.ox.ac.uk;
Hasan Yardimci, hasan.yardimci@crick.ac.uk

DOI: 10.1126/science.adf0224

The PDF file includes:

Materials and Methods
Figs. S1 to S16
Table S1
References

Other Supplementary Material for this manuscript includes the following:

MDAR Reproducibility Checklist
Movies S1 to S7

Materials and Methods

Protein expression, purification and labeling

Fen1-mKikGR. Purification was carried out as previously described (*17*).

Cohesin-trimer and tetramer (Halo-JF646/JF549).

Construction of the expression vectors for cohesin tetramer was described previously (*10*). To generate vectors containing cohesin trimer, *Xenopus laevis* SMC1 and SMC3 with a C-terminal Halo and 2X Flag tags were cloned into MultiBac vectors (pACEbac1). *Xenopus laevis* RAD21 with a C-terminal His8 tag was cloned into pDIC plasmid. The pACEbac1 XSMC1 XSMC3-Halo-Flag and pIDC XRAD21-8xHis were then combined by a Cre recombinase reaction (New England Biolabs). To generate the baculoviruses, DNAs were first transformed into DH10Bac (Thermo Fisher) cells and bacmids containing the expression vector screened for by blue-white selection. Bacmid DNA was then extracted and 2 μg of bacmid DNA was transfected into 2 ml *S. frugiperda* Sf9 cells (Thermo Fisher) at a cell density of 1×10^6 cells ml^{-1} using FuGENE HD reagent (Promega), grown in Sf900 II SFM media (Thermo Fisher). These were then incubated at 27°C for 5 days to create P1 virus. P2 virus was then amplified by infecting 50 ml Sf9 cells at a density of 2×10^6 cells ml^{-1} with 500 μl P1 virus and incubating in the dark at 27°C for 3 days with shaking at 100 rpm.F

Typically, proteins were expressed by adding 5 ml P2 virus to 500 ml Sf9 cells at a density of 2×10^6 cells ml^{-1} and incubating in the dark at 27°C for 2 days with shaking at 100 rpm. Cells were then harvested by centrifugation at 1000*g*, washed with PBS, frozen in liquid nitrogen and stored at −80°C. All subsequent steps were performed on ice or at 4°C. Cells were lysed by thawing and dounce homogenizing in buffer A500 (25 mM HEPES-KOH pH 7.5, 500 mM KCl, 5% v/v glycerol), 20 mM β-mercaptoethanol, 0.05% v/v Tween-20, 0.5 mg/ mL PMSF and complete protease inhibitor (Roche). After lysis, an equal volume of buffer A0 (buffer A500 lacking KCl) was added to the lysate and centrifuged at 75,000*g* for 40 min. The clarified lysate was filtered through a 0.45 μm filter and cohesin was purified in the AKTA system using a 5 mL (HisTrap) TALON column (VWR). The column was washed with 20 column volumes of buffer B (20 mM Tris PH7.5, 250 mM KCL, 5% glycerol) and eluted over a linear gradient of 0-500 mM imidazole. The peak fractions containing cohesin were pooled and incubated with anti-FLAG-M2 resin (Sigma) and 10nM JF646 or JF549 HaloTag ligand (a kind gift from Luke Lavis (*36*)) for 3 hours at 4°C in the dark. The beads were pelleted and washed with 5X EB buffer (100 mM KCl, 2.5 mM $MgCl_2$ and 50 mM HEPES-KOH pH 7.5) and eluted in EB buffer with FLAG peptide containing 5% glycerol and 5 mM DTT. Finally, proteins were purified via size exclusion chromatography using a Superose 6 increase 10/300 GL column (VWR). Peak fractions were collected, concentrated to typically 3 μM and stored in aliquots at −80°C.

SA1. Xenopus laevis SA1 was cloned into pACEbac1 vector with a C-Terminal His8 tag. The P1 and P2 viruses were generated as described above. SA1 was expressed by adding 5 ml P2 virus to 500 ml Sf9 cells at a density of 2×10^6 cells ml^{-1} and incubating in the dark at 27°C for 2 days with shaking at 100 rpm. Cells were then harvested by centrifugation at 1000*g*, washed with PBS, and then frozen in liquid nitrogen and stored at −80°C. All subsequent steps were performed on ice or at 4°C. Cells were lysed by thawing and dounce homogenizing in buffer A500 (25 mM HEPES-KOH pH 7.5, 500 mM KCl, 5% v/v glycerol), 20 mM β-mercaptoethanol, 0.05% v/v Tween-20, 0.5 mg/ mL PMSF and complete protease inhibitor (Roche). After lysis, an equal volume of buffer A0 (buffer A500 lacking KCl) was added to the lysate and centrifuged at

75,000*g* for 40 min. The clarified lysate was filtered through a 0.45 µm filter and cohesin was purified in the AKTA system using a 5 mL (HisTrap) TALON column (VWR). The column was washed with 20 column volumes of buffer B (20 mM Tris PH7.5, 250 mM KCL, 5% glycerol) and eluted over a linear gradient of 0-500 mM imidazole. The peak fractions containing SA1 were pooled, concentrated to 1 ml final volume, and purified via size exclusion chromatography using a Superose 6 increase 10/300 GL column (VWR). Peak fractions were collected, concentrated to typically 3 µM and stored in aliquots at −80°C.

NIPBL-C. HsNIPBL-C (residues 1163-2804) was cloned into pAECbac1 vector with an N-terminal His6 and a C-terminal Flag tag. P1 and P2 viruses were generated as described above. NIPBL-C was expressed by adding 10 ml P2 virus to 500 ml Sf9 cells at a density of 2×10^6 cells ml^{-1} and incubating in the dark at 27°C for 2 days with shaking at 100 rpm. Cells were then harvested by centrifugation at 1000*g*, washed with PBS, and then frozen in liquid nitrogen and stored at −80°C. All subsequent steps were performed on ice or at 4°C. Cells were lysed by thawing and dounce homogenizing in buffer A500 (25 mM HEPES-KOH pH 7.5, 500 mM KCl, 5% v/v glycerol), 20 mM β-mercaptoethanol, 0.05% v/v Tween-20, 0.5 mg/ mL PMSF and complete protease inhibitor (Roche). After lysis, an equal volume of buffer A0 (buffer A500 lacking KCl) was added to the lysate and centrifuged at 75,000*g* for 40 min. The clarified lysate was filtered through a 0.45 µm filter and cohesin was purified in the AKTA system using a 5 mL (HisTrap) TALON column (VWR). The column was washed with 20 column volumes of buffer B (20 mM Tris PH7.5, 250 mM KCL, 5% glycerol) and eluted over a linear gradient of 0-500 mM imidazole. The peak fractions containing NIPBL were pooled and incubated with anti-FLAG-M2 resin (Sigma) for 3 hours at 4°C. The beads were pelleted and washed with 5X EB buffer (100 mM KCl, 2.5 mM $MgCl_2$ and 50 mM HEPES-KOH pH 7.5) and eluted in EB buffer with Flag peptide containing 5% glycerol and 5 mM DTT. The protein was concentrated to typically 1 µM and stored in aliquots at −80°C.

GINS. Purification was adapted from a previously described protocol (*24*). The MultiBac vectors pFL-Psf1/Psf2 and pSPL-Sld5/Psf3-LPETG-Flag were cloned using codon-optimized GINS sequences synthezised by GeneArt (Sigma). Sequences encoding a linker (GGGGSGGGGS), a Sortase labeling tag (LPETG) and a Flag epitope (DYKDDDDK) were included to be incorporated at the C-terminus of Psf3. pFL-Psf1/Psf2 and pSPL-Sld5/Psf3 plasmids were combined by Cre-Lox recombination, and the fused pFL/pSPL plasmid transformed into DH10MultiBac cells to create a bacmid. Sf9 insect cells were transfected and multiple rounds of virus amplification were performed. Hi5 insect cells (1×10^6 cells/mL) were infected for GINS expression and were harvested after 48 hours.

Purification steps were performed at 4°C. A pellet from 1 L cells was resuspended in 40 mL lysis buffer (20 mM Tris pH 8, 500 mM NaCl, 0.1% NP40, 10% glycerol, 2 mM β-mercaptoethanol, 1 x EDTA-free protease inhibitor tablet (Roche)) and sonicated for 2 min. The lysate was cleared with centrifugation at 30,000*g* for 30 min, and the supernatant was incubated with 3 mL pre-washed Anti-FLAG M2 affinity gel (Sigma) for 2 hours. The resin was washed 2 x 15 mL wash buffer (20 mM Tris pH 8, 500 mM NaCl, 0.1% NP40, 10% glycerol and 2 mM β-mercaptoethanol). Protein was eluted with 2 x 5 mL wash buffer/333 µg/mL Flag peptide. The Flag resin eluate was loaded onto a MonoQ 5/50 GL (Cytiva) column prequilibriated with MonoQ-100 (20 mM Tris pH 7.5, 100 mM NaCl, 10% glycerol and 1 mM DTT). GINS was eluted using a linear gradient of MonoQ-700 (20 mM Tris pH 7.5, 700 mM NaCl, 10% glycerol and 1 mM DTT).

For fluorescent labeling, 3 volumes of a mixture containing 4 nmol GINS, 1 nmol Sortase A and 100 nmol fluorescent peptide (NH2-GGGHHHHHHC(*)-COOH, where *=LD555, LD655 or AF647, conjugated using maleimide-thiol reaction) was added to 1 volume of labeling buffer (80 mM HEPES pH 7.5, 600 mM NaCl, 40% glycerol, 20 mM $CaCl_2$ and 4 mM DTT). After an overnight reaction, GINS was purified using a Sepharose 200 10/300 GL size exclusion column equilibrated in GF buffer (20 mM Tris pH 7.5, 150 mM NaCl, 10% glycerol and 1 mM DTT). To selectively purify labeled GINS containing polyhistidine, peak fractions were supplemented with 10 mM imidazole and 10 µg/mL aprotinin/leupeptin and bound to NiNTA beads (Qiagen) for 2 hours. Beads were washed with GF buffer/10 mM imidazole then GINS eluted with GF buffer/500 mM imidazole. The protein was dialyzed in GF buffer overnight before storage at -80°C.

LacI-AF488. Labeled LacI was purified essentially as described (*37*). Plasmids containing LacI with a C-terminal HHHHHHC ('LacI-Far', a gift from Sebastian Deindl) were transformed into BL21 cells. Expression was induced for 3 hours in 1 L of cells using 0.2% L-rhamnose. Cell pellets were resuspended in 20 mL buffer A (20 mM phosphate pH 7.4, 500 mM NaCl, 5 mM β-mercaptoethanol, 20 mM imidazole) with 1 EDTA-free protease inhibitor tablet (Roche) and supplemented with 10 µg/mL lysozyme and 25 U/mL benzonase. After sonication for 2 min total, lysate was cleared by centrifugation at 20,000*g* for 20 min and passed through a 0.45 µm filter. LacI was bound to a 5 mL HisTrap HP column equilibrated in buffer A and eluted with a linear gradient of buffer B (20 mM phosphate pH 7.4, 500 mM NaCl, 5 mM β-mercaptoethanol, 500 mM imidazole). Protein was buffer exchanged into phosphate buffered saline (PBS) containing 10% glycerol using a spin concentrator (10 kDa MWCO, Millipore).

For labeling, 1 mg Alexa Flour 488 C_5 Maleimide (ThermoFisher) was dissolved in 50 µL DMSO then mixed with ~0.5 µmol LacI in degassed PBS containing 10% glycerol and 500 µM TCEP. After 90 min at room temperature, the reaction was quenched with 5 mM β-mercaptoethanol. LacI-AF488 was purified with a 5 mL HisTrap HP column (as described above), before peak fractions were dialyzed into PBS with 500 mM NaCl overnight. LacI-AF488 was diluted 1:1 with glycerol before storing aliquots at -80°C.

<u>Xenopus laevis egg extract preparation and immunodepletion</u>

Xenopus laevis egg extract preparation. Animal husbandry, injections and egg collection were performed by the Francis Crick Institute Aquatics Facility. Extracts and sperm chromatin were prepared as described (*38*) and aliquots were stored at -80°C.

Cohesin immunodepletion. For depletions, rProtein A Sepharose Fast Flow (PAS, Cytiva) beads were extensively washed with PBS before antibody binding. After antibody binding, beads were washed 3 times with PBS and 5 times with Egg Lysis Buffer (ELB, 50 mM HEPES pH 7.7, 2.5 mM $MgCl_2$, 50 mM KCl), before transferring to siliconized microcentrifuge tubes. To recover extracts from PAS beads between rounds of depletion, the extract/bead mixture was applied to a homemade nitex filter (*38*) and centrifuged at 2,800*g* for 40 seconds.

SMC1 and SMC3 antibodies were a kind gift from Vincenzo Costanzo. Both antibodies were raised in rabbits immunized with peptides: anti-SMC1 with DLTKYPDANPNPND and anti-SMC3 with EQAKDFVEDDTTHG. A cysteine was added to the N-terminus of both peptides, and the modified peptides were used for immunoaffinity purification according to manufacturer's instructions (SulfoLink Immobilization Kit for Peptides, ThermoFisher). 72 µL of PAS beads were incubated overnight with 100 µg purified SMC1 antibody and 100 µg purified SMC3 antibody. 1

µL 0.5 mg/mL nocodazole was added to 60 µL HSS and 60 µL NPE supplemented with DTT to a final concentration of 10 mM. HSS and NPE were mixed and added to 24 µL of anti-SMC1/anti-SMC3 PAS beads, for 3 rounds of depletion for 45 min at 4°C.

For cohesin depletions used for sister DNA collapse experiments and measuring bulk DNA replication timing, the same ratios of SMC1/SMC3 antibodies to beads were used. Mock depletions used PAS beads washed with ELB only. 60 µL HSS was added to 10 µL beads, for two rounds of depletion. A mixture of 90 µL HSS with 90 µL NPE was added to 30 µL PAS beads, for three rounds of depletion. For NPE depletion alone for bulk replication, 32 µL NPE was added to 6 µL PAS beads for three rounds of depletion. Depleted extract was aliquoted and frozen at-80°C before use.

GINS immunodepletion. Rabbits were immunized with *Xenopus laevis* GINS purified from insect cells. Anti-GINS antibody was affinity purified using protein A-sepharose (Covalab). For bulk replication assays, HSS and NPE were separately depleted. 300 µL purified anti-GINS antibody (3.5 mg/mL) was incubated overnight with 120 µL PAS beads. 180 µL HSS was supplemented with 3 µL 0.5 mg/mL nocodazole and added to 30 µL anti-GINS PAS beads for 2 rounds of depletion for 45 min at 4°C. 90 µL of NPE supplemented with DTT to a final concentration of 10 mM was added to 20 µL anti-GINS PAS beads for 3 rounds of depletion. 15 µL ΔGINS extracts were stored at -80°C before use.

For single-molecule replication assays, a mixture of HSS and NPE was depleted. 150 µL purified anti-GINS antibody (3.5 mg/mL) was incubated with 40.5 µL PAS beads overnight. 13.5 µL of washed beads were used for each round of depletion. 22.5 µL of HSS was supplemented with 0.35 µL 0.5 mg/mL nocodazole and mixed with 45 µL of NPE supplemented with DTT to a final concentration of 10 mM. The extract mixture was depleted for 3 rounds of 1 hour, before storing 15 µL aliquots of HSS/NPEΔGINS at -80°C.

Bulk DNA replication assays. HSS and NPE were activated (see "Cohesin loading onto tethered DNAs" below), with NPE supplemented with [α^{32}P]-dATP. 10 ng/µL pBlueScript was added to HSS and licensing was performed for 30 min. 1 volume licensing mixture was mixed with 1 volume NPE and 1 volume ELB to initiate DNA replication. For experiments with GINS-depleted extracts, 1 volume licensing mixture was added to 1 volume 1.5 µM GINS or 0.3 µM AF647-GINS diluted in ELB, then 1 volume NPE was added to begin DNA replication. Reactions were stopped with SDS, treated with proteinase K, and separated on a 0.8% agarose gel before visualization by autoradiography.

Preparation of DNA substrates

mBiotin-λ-mDigoxigenin. PCR reactions were used to incorporate biotin- or digoxigenin-modified nucleotides into handles ligated to the ends of λ DNA. Two PCR reactions using GoTaq G2 PCR mix (Promega) and a pUC19 template were set up: PCR-Dig with oGC101/oGC102 primers and 25 µM digoxigenin-11-dUTP (Roche), and PCR-Bio with oGC101/oGC103 primers and 25 µM biotin-16-dUTP (Enzo). The products were isolated with a PCR purification kit (Qiagen), nicked with Nt.BspQI (NEB) and heated to 65°C to create 12 bp ssDNA ends complementary to λ DNA ends. Before cooling, PCR-Bio was mixed with oGC104 and PCR-Dig was mixed with oGC105 to prevent reannealing. The handles were separated on a 1.5% agarose gel and purified using a gel purification kit (Qiagen). λ DNA was phosphorylated with T4 polynucleotide kinase (NEB), ligated to PCR-Bio/Nt.BspQI with T4 DNA ligase (NEB) and purified from a 0.5% agarose gel

by electroelution. The product was ligated to PCR-Dig/Nt.BspQI and purified once more, before aliquots were stored at -20°C and freeze-thaw cycles were avoided.

List of oligonucleotides used for making mBiotin-λ-mDigoxigenin substrates
oGC101 ATGCCGGGAGCAGACAAGCCCGTC
oGC102 GGGCGGCGACCTGGAAGAGCAGCTGGCACGACAGGTTTCCCG
oGC103 AGGTCGCCGCCCGGAAGAGCAGCTGGCACGACAGGTTTCCCG
oGC104 AGGTCGCCGCCC
oGC105 GGGCGGCGACCT

Biotin-λ-Biotin. Doubly biotinylated DNA was prepared as previously described (*22*). 10 μg λ DNA (NEB) was added to a 50 μL reaction with 80 μM biotin-dCTP (Invitrogen), 80 μM biotin-dATP (Invitrogen), 100 μM dTTP and 100 μM dGTP, then heated to 65°C for 5 min. 2.5 U Klenow polymerase (NEB) was added and the reaction was incubated for 30 min at 37°C for 30 min, then at 70°C for 15 min. DNA was purified by electroelution from a 0.5% TBE gel, then dialyzed into 10 mM Tris pH 7.5 and 1 mM EDTA. Aliquots were stored at -20°C and freeze-thaw cycles were avoided.

Biotin-LacO-23kb-LacO-Biotin. pHY42, a 17.3 kb plasmid containing a 48x*lacO* array (~1.5 kb), was digested with BsiWI-HF restriction enzyme. The 4 bp overhangs formed were filled in with biotin-11-dGTP (Jena Bioscience), biotin-16-dUTP (Roche), biotin-14-dCTP (Invitrogen) and biotin-14-dATP (Invitrogen), each added to a final concentration of 50 μM in a reaction with ~6 μg DNA and 15 U Klenow polymerase. The reaction was buffer exchanged in a Microspin G-50 Column (Cytiva) before treatment with Quick Calf Intestinal Phosphatase (CIP, NEB). The plasmid was digested with AgeI, creating a 12.9 kb fragment that was separated from a smaller 4.4 kb fragment on a 0.6% agarose gel, then purified by electroelution. The fragment was self-ligated with T4 DNA ligase (NEB) to create a 25.8kb linear DNA with a 48x*lacO* array and 3'-biotins at either end. The DNA substrate was purified from a 0.5% agarose gel by electroelution.

Coverslip functionalization, microfluidic flow channel preparation and DNA tethering

Coverslips were functionalized and microfluidic flow channels were prepared essentially as previously described (*18*). 24 x 60 mm glass coverslips (VWR) were sonicated in ethanol for 30 min and 1 M KOH for 30 min, with rinsing in water performed between sonications. This was repeated once before plasma cleaning and silane treatment in 2% v/v 3-aminopropyltriethoxysilane (in acetone) for 2 min. After rinsing in water, 75 mg mPEG-SVA (MW 5,000, Laysan Bio) and 2 mg biotin-PEG (MW 5,000, Laysan Bio) dissolved in 500 μL 100 mM $NaHCO_3$ were placed between two coverslips and incubated for 3 hours. Coverslips were rinsed and stored under vacuum.

Microfluidic flow channels were assembled using a cut glass slide with holes drilled for PE20 inlet and PE60 outlet (Intramedic) polyethylene tubing. Double-sided tape (AR90880, Adhesive Research) was cut and sandwiched between the coverslip and glass slide, creating a flow channel sealed with epoxy resin.

Flow channel outlet tubing was connected to a syringe pump (Harvard Apparatus) and the flow channel was washed in blocking buffer (20 mM Tris pH 7.5, 50 mM NaCl, 2 mM EDTA, 0.2 mg/mL BSA). 0.2 mg/mL streptavidin in blocking buffer was incubated in the flow channel for 10 min before DNA tethering. mBiotin-λ-mDigoxigenin DNAs were diluted to <1 ng/μL in blocking

buffer and incubated for up to 30 min to tether the biotinylated end then washed. Single-tethered DNA was stretched with blocking buffer containing 1 μg/mL biotinylated anti-digoxigenin (Perkin Elmer) at 100 μL/min flow rate. Biotin-λ-biotin and Biotin-LacO-23kb-LacO-Biotin DNAs, at a concentration <1 ng/μL, were bound to surfaces at 100 μL/min flow rate for between 2 and 10 min. Tethered DNAs were stained with 5 nM SYTOX Orange (ThermoFisher) to check DNA concentration and end-to-end distances.

Cohesin loading on chromatin

HSS extracts were supplemented with either EB buffer (100 mM KCl, 2.5 mM $MgCl_2$ and 50 mM HEPES-KOH pH 7.5) or with 60nM Geminin and incubated at 23 °C for 10 min. This was followed by addition of 400nM recombinant cohesin and sperm chromatin. Reactions were incubated at 23 °C for 60 min. To isolate HSS extract assembled chromatin, samples were diluted in ten volumes of EB buffer containing 0.25% Nonidet P-40 and centrifuged through a 30% sucrose (in EB) layer at 10,000 rpm for 5 min at 4°C using a HB-6 rotor (Sorvall), washed three times with 500 μL EB buffer and centrifuged at 10,000 rpm for 1 min. The pellet was resuspended in Laemmli loading buffer and the proteins resolved by either 4%–15%, 7.5% or 10% SDS-PAGE and analyzed by western blotting with specific antibodies as indicated (anti-Smc3, anti-MCM7, and anti-ORC2 antibodies were described before (*10*). Smc3 was detected using IRDye 680RD Goat-Anti Rabbit antibody (Licor)).

Cohesin loading onto tethered DNAs and DNA replication with labeled Fen1

Prior to DNA replication with Fen1-mKikGR-labeled replication bubbles, labeled cohesin was loaded onto mBiotin-λ-mDigoxigenin or Biotin-λ-Biotin template DNAs during replication licensing. An ATP regeneration mixture was assembled with 5 μL 0.2 M ATP, 10 μL 1 M phosphocreatine and 0.5 μL 25 U/mL creatine phosphokinase. ELBS buffer used to dilute extracts was made from 30 μL ELB containing 0.25 M sucrose and supplemented with 1 μL ATP regeneration mixture. 33 μL HSS was mixed with 0.5 μL 0.5 mg/mL nocodazole and 1 μL ATP regeneration mixture. 16 μL NPE was mixed with 0.5 μL ATP regeneration mixture, and both HSS and NPE extracts were centrifuged for 5 min at 16,000*g* before use. A licensing mixture was assembled containing 15 μL HSS, 15 μL ELBS and 0.75 μL 400 ng/μL oligonucleotide duplex (*18*), and JF646-labeled cohesin tetramer was added to a final concentration of 500 nM. DNA was incubated with licensing mixture for 25 min. A mixture of 15 μL NPE, 15 μL HSS and 15 μL ELBS supplemented with 5 ng/μL pBlueScript and 2.5 μM Fen1-mKikGR was split into two, with half the mixture infused into flow channels to initiate DNA replication. The remainder was supplemented with 0.1 μg/mL $p27^{kip}$ and added to flow channels after between 2 and 10 min to limit further origin firing. For experiments with cohesin-depleted extract, 30 μL of cohesin-depleted HSS/NPE was mixed with 1 μL ATP regeneration mixture. The depleted extract was then mixed with 15 μL ELBS and supplemented with pBlueScript and Fen1-mKikGR as described above. For imaging in high salt buffer after replication, ELB containing 500 mM KCl was infused into the flow channel.

DNA replication with labeled GINS

Single-molecule replication assay with GINS-depleted extracts. Biotin-λ-Biotin template DNAs were used for reactions with labeled GINS. HSS and NPE were prepared as above. 15 μL GINS-depleted HSS/NPE was mixed with 0.5 μL ATP regeneration mixture. 10 μL HSS, 10 μL ELBS and 0.5 μL 400 ng/μL oligonucleotide duplex were mixed and infused into flow channels for 10 min of licensing. The channel was washed with 60 μL ELB supplemented with 1 mg/mL BSA and 0.5 mg/mL casein. A mixture of 15 μL GINS-depleted HSS/NPE, 5 μL ELBS, 4 μL LD655-

/LD555-GINS (~2-5 μM), 0.5 μL 150 ng/μL pBlueScript and 0.2-1 μL 0.5 mg/mL cyclin A2 (Abcam) was infused into the flow channel for 2-5 min. To prevent further origin firing and wash away excess fluorescent GINS, a mixture of 15 μL NPE, 22 μL HSS, 15 μL ELBS, 2 μL 0.1 μg/mL $p27^{kip}$ and 1.2 μL 150 ng/μL pBlueScript, supplemented with ~2.5 μM Fen1-mKikGR when required, is added to the flow channel. Replication extracts were supplemented with 200 μM NMS-873 (Sigma) to inhibit p97 enzyme.

To load cohesin on DNA before replication with labeled GINS, cohesin was loaded onto DNAs in buffer prior to licensing. 1 μL 2 μM JF646-cohesin trimer (SMC1/SMC3/RAD21), 1.5 μL 3 μM SA1 and 1.5 μL 3 μM NIPBLc were mixed and incubated on ice for 10 min. Cohesin buffer was made from ELB supplemented with 1 mg/mL BSA, 5 mM DTT, 0.002% Tween-20 and 5% glycerol, and this was used to wash DNAs tethered in flow channels. 1 μL cohesin/loader mixture was diluted in 200 μL cohesin buffer containing 3 mM ATP, and incubated with tethered DNAs for 10-15 min. The flow channel was washed with cohesin buffer before replication with labeled GINS as described above.

<u>Experiment to monitor sister DNA collapse</u>
For experiments with labeled cohesin pre-loaded onto DNAs, JF549-cohesin was loaded onto tethered *Biotin-LacO-23kb-LacO-Biotin* DNAs in buffer as described above. HSS and NPE were prepared as above. Licensing was performed with a mixture of 15 μL HSS, 5 μL ELBS and 0.75-1.5 μL 400 ng/μL oligonucleotide duplex for 10 min. 30 μL NPE, 30 μL HSS, 30 μL ELBS and 2-4 μL 150 ng/μL pBlueScript were mixed and split into 3 x 30 μL reaction mixtures. The first firing extract was supplemented with 2-3 μL 0.5 mg/mL cyclin A2, 1.5-1.8 μL 1 mM AF647-dUTP (Jena BioScience) and 0.75 μL 25 μM LacI-AF488 and incubated in the flow channel for between 10 and 15 min. The second firing mixture was infused into the flow channel to remove excess AF647-dUTP and LacI-AF488 for 3 min. A third firing mixture was supplemented with 1.5 μL 1 M IPTG, to remove LacI from the *lacO* sequences at the ends of DNA, was infused into the flow channel.

To compare the length of time collapsed sister DNAs survived in differently depleted extracts, experiments were performed as described above. Licensing mixture was supplemented with 1.5 μL 400 ng/μL oligonucleotide duplex and 3 μL 0.2 mg/mL CDT1 (*39*) to ensure maximal pre-RC assembly. To remove as much fluorescent nucleotide as possible, the volume of the third firing mixture was increased to 60 μL and infused at a 1.5 μL/min flow rate for 40 min. Images were taken for 60 min for each type of depleted extract.

For comparing sister DNA collapse ±p97i, p97i (NMS-873) was diluted 100 times to a final concentration 200 μM, or the equivalent volume of DMSO was added. JF646-Cohesin was pre-loaded onto DNAs, before licensing and incubation in the first firing mixture for 30 minutes. Washing with the second and third firing mixtures was performed as described above.

<u>SDS washing after sister DNA collapse</u>
Biotin-LacO-23kb-LacO-Biotin DNAs were replicated as described above. After infusing the third replication firing mixture into the flow channel, nascent DNAs with incorporated AF647-dUTP were imaged for 8 minutes to visualize sister DNA collapse. SDS-containing buffer (20 mM Tris pH7.5, 50 mM NaCl, 12 mM EDTA, 0.1% SDS) was then continuously infused into the flow channel, whilst DNAs labeled with AF647-dUTP were imaged.

<u>Image acquisition and analysis</u>

Image acquisition and processing. Images were acquired using a Nikon Eclipse Ti microscope as previously described (*22*). A 5x5 or 6x6 grid of field of view was imaged during DNA replication, typically with a lapse time of 60-90 seconds. Images were initially processed in NIS Elements, with the "Advanced Denoising" function used with a denoising power of 5 for each channel. In some cases, a rolling ball background correction (radius 0.96 μm) was used for background subtraction. Images were corrected for drift using the align function. Fiji was then used to rotate and crop regions of interest, with a width of 5-7 pixels, and to create kymograms using the "Make Montage" function.

Measuring fork speeds. Average DNA lengths were measured for each experiment in BB supplemented with 5 nM SYTOX Orange without any flow. To calculate fork speeds, Fen1-mKikGR replication bubble growth was measured during a period of constant fork movement and averaged between 2 diverging replisomes. The rates of individual labeled replisome molecules were measured individually.

Defining cohesin fates after collision with the replication forks. Cohesin-fork encounter was defined as colocalization of cohesin signal (diffraction-limited spot) with the tip of Fen1-mKikGR-decorated replication bubble. Cohesin removal was marked by the loss of cohesin fluorescence in the next time frame upon fork encounter. Cohesin transfer was assigned when, upon fork encounter, cohesin signal was incorporated into the replication bubble and could be followed for at least two subsequent time frames (2 min). Cohesin sliding was determined by a unified cohesin-fork movement over at least 3 pixels. Replication fork stalling was assigned if a fork movement was arrested by a static (within 2 pixels) cohesin fluorescence for at least three time frames (3 min).

Defining cohesin fates after collision by labeled replisomes. When the replisome and cohesin co-localize in a diffraction limited spot moving >2 pixels in <5 min, cohesin sliding was scored. When co-localizing replisomes and cohesin do not move >2 pixels in >5 min, stalling was scored. When there was no detectable change in replisome speed during co-localization with cohesin, and cohesin fluorescence is lost without moving >2 pixels, the event was defined as removal. When a replisome and cohesin co-localize for <5 min, and cohesin remains in the same spot whilst the replisome moves >2 pixels away, a transfer event was scored. Cohesin fates during replication termination were defined similarly. Only events where both converging replisomes were labeled, at least one of these converging replisomes is associated with a sliding cohesin and where the replisome is disassembled after convergence were included. With p97i, replisomes were considered to have bypassed one another if moving >2 pixels apart in <15 minutes.

Photobleaching assays. For comparing loss of cohesin fluorescence in non-replicating extract versus in buffer, JF646-cohesin was loaded on mBiotin-λ-mDigoxigenin DNAs in cohesin buffer for 10 min, as described above. A non-replicating extract mixture (15 μL HSS, 15 μL NPE, 15 μL ELBS, 2 μL 150 ng/μL pBlueScript, 2 μL 2.5 mg/mL p27kip), or 50 μL cohesin buffer was used to wash flow cells. Images of cohesin were collected every 60 s over a 6x6 grid for 30 min ("slow imaging"), with the same laser settings routinely used for JF646-cohesin. Alternatively, to measure the rate of photobleaching, after washing with buffer, single fields of view were imaged with a 100 ms lapse time for 60 frames ("fast imaging"). After denoising, background correction, and alignment, average fluorescence intensities were measured in 76x6 pixel boxes around cohesin-bound DNAs for each dataset. Fluorescence intensities were normalized to the local background at each time point, measured using a 3x3 pixel box. The fast imaging data were fit to a one-phase

exponential decay curve in GraphPad Prism, which was used to correct for photobleaching in the slow imaging datasets. The corrected slow imaging datasets were fit to a one-phase exponential decay curve.

For photobleaching cohesin molecules remaining on DNA after replication termination, *Biotin-LacO-23kb-LacO-Biotin* DNAs were replicated essentially as described in "Experiment to monitor sister DNA collapse". JF646-cohesin and AF555-dUTP (Jena BioScience) were used instead of JF549-cohesin and AF647-dUTP, respectively. After initial origin firing for 20 min, the flow cell was washed with 70 µL of a mixture of NPE, HSS and ELBS, supplemented with 6 ng/µL pBlueScript and 830 nM LacI-AF488, to remove excess fluorescent nucleotide whilst maintaining fork arrest at DNA ends. 5-10 fields of view were imaged once using 561 nm laser excitation then imaged 100 times with 640 nm laser excitation. The 640 nm laser power was increased 10-fold over the power used during normal cohesin imaging. Images of AF555-dUTP were corrected with a rolling ball correction, then fully replicated DNAs were cropped with 55x6 pixel boxes. A 5x5 pixel box was cropped around cohesins found in the center of replicated DNAs, and the fluorescence intensities at each timepoint during 100 frames of imaging were extracted. Autostepfinder (set with ItRange = 5, AccThreshold = 0.3) (*40*) was used to count photobleaching steps, with excessively noisy data excluded.

For measuring cohesin stability after completion of DNA replication, replication of DNAs pre-loaded with JF646-cohesin was performed as described above, with LacI-AF488 used to pause forks and AF555-dUTP used to label nascent DNA. A 6x6 grid was imaged with alternating 561 nm and 640 nm laser excitation every 5 min for a total of 60 min. Fully replicated DNAs were selected, cropped, and retention time of cohesin at the center of DNAs was measured.

Sister DNA collapse experiments. Cohesin and the collapsed sister DNA were considered to be colocalized when <2 pixels apart. We assumed the probability of cohesin binding exclusively to either strand was equal. 15-27% of cohesin colocalized with the stretched sister DNA so we assumed a further 15-27% of cohesin colocalized exclusively with one collapsed sister DNA, therefore inferring that 46-70% of cohesin bound to both sister DNA.

To compare the length of time collapsed sister DNAs survived in differently depleted extracts, only events where the entire DNA was replicated and both sister DNAs collapsed were considered. Events where collapsed sister DNAs remained together until the end of 60-min imaging were also included.

For analyzing the fate of colocalizing sister DNAs during SDS-washing, DNAs were selected when DNAs were fully replicated (as shown with AF647-dUTP signal), and where sister DNA colocalization was observed before SDS washing. SDS-mediated strand separation was scored when DNAs separated into two clear sister DNAs during the first two minutes of imaging in SDS-containing buffer.

Yeast cell culture. All strains are derivatives of W303 (K699). Strain numbers and relevant genotypes of the strains used are listed in Supplementary Table 1. Wild type and Cdc48-AID cells were cultured at 25°C in YEP medium with 2% glucose. To arrest the cells in G1, α-factor was added to a final concentration of 2 mg/L, every 30 min for 3 hr. 5 µM indole-3-acetic acid (IAA, Santa Cruz) was added to the culture 90 min before release from the G1 arrest. Cells were

released from G1 arrest by filtration wherein cells were captured on 1.2 µm membrane (Whatman GE Healthcare), washed with 1 L YEPD and resuspended in YEPD medium containing 10 µg/mL nocodazole (Sigma) and 5 µM IAA. Wild Type, *dia2*Δ and *dia2-13A* mutants were grown asynchronously at 25°C in YEP medium with 2% glucose.

To test the stability of linear mini-chromosomes, wild type and *dia2*Δ mutant strains were grown for 40 generation at 25°C in YEP medium with 2% glucose. Cells were serially diluted and plated onto YEP plates with 2% glucose and grown at 25°C for 2-3 days. The total number of colonies on the plates were counted and the plates were replicated onto SC-URA and SC-MET plates and grown at 25°C for 2-3 days. The number of cells growing on YPD, SC-URA and SC-MET plates were counted and the % cells retaining the respective mini-chromosomes was calculated (Number of colonies of the respective selective medium/Number of colonies on YPD) × 100.

To assess depletion of the Cdc48-AID protein. Cells grown as described above were washed with 2 × 2 mL ice-cold PBS containing 5 mM DTT, resuspended in 500 µL lysis buffer (25 mM Hepes pH 8.0, 50 mM KCl, 50 mM $MgSO_4$, 10 mM trisodium citrate, 25 mM sodium sulfite, 0.25% triton-X, freshly supplemented with Roche Complete Protease Inhibitors (2X) and PMSF (1 mM), lysed in a FastPrep-24 (MP Biomedicals) for 3 × 1 min at 6.5 m/s with 500 µl of acid-washed glass beads (425–600 µm, Sigma) and lysates cleared (5 min, 12 kg). Protein concentrations were measured using Bradford assay and whole cell lysates were resolved in NuPAGE 3–8% or 4–12% gradient gels (ThermoFisher Scientific) and transferred onto PVDF membranes using the Trans-blot Turbo transfer system (BioRad). The membrane was probed anti-PK antibody (AbD Serotec). For visualization, the membrane was incubated with Immobilon Western Chemiluminescent HRP substrate (Millipore) before detection using an ODYSSEY Fc Imaging System (LI-COR).

Sister chromatid cohesion assay in S.cerevisiae. Cells were fixed with 4% formaldehyde for 45 min at room temperature, washed 2X with PBS sorbitol (1xPBS, 1M Sorbitol) and stored at 4°C. In situ immunofluorescence to detect MYC (Sc-40 Anti MYC antibody from Santacruz) tagged Securin and GFP marked *URA3* loci was carried out as described in (*41*). Briefly, the fixed cells were spheroplasted by treatment with Zymolyase (100T) for 30 min at 30°C. The spehroplasts were adhered onto a polylysine coated slide and permeabilized by incubation with 1% NP-40 for 5 min. The slides were blocked with PBS containing 1% BSA and incubated overnight with PBS/1% BSA/anti MYC antibody at 4°C. The slides were washed 10 rounds with PBS/1% BSA and treated with fluorescently labeled secondary antibody for 2 hr at room temperature. The slides were mounted with DAPI containing mounting medium and observed with a Zeiss Axio Imager.Z1 microscope (63 × objective, NA = 1.40) equipped with a coolSNAP HQ camera. All experiments were repeated 3 times, and at least 150 large budded mitotic cells were analyzed for each repeat. The GFP dots were scored by double blinding.

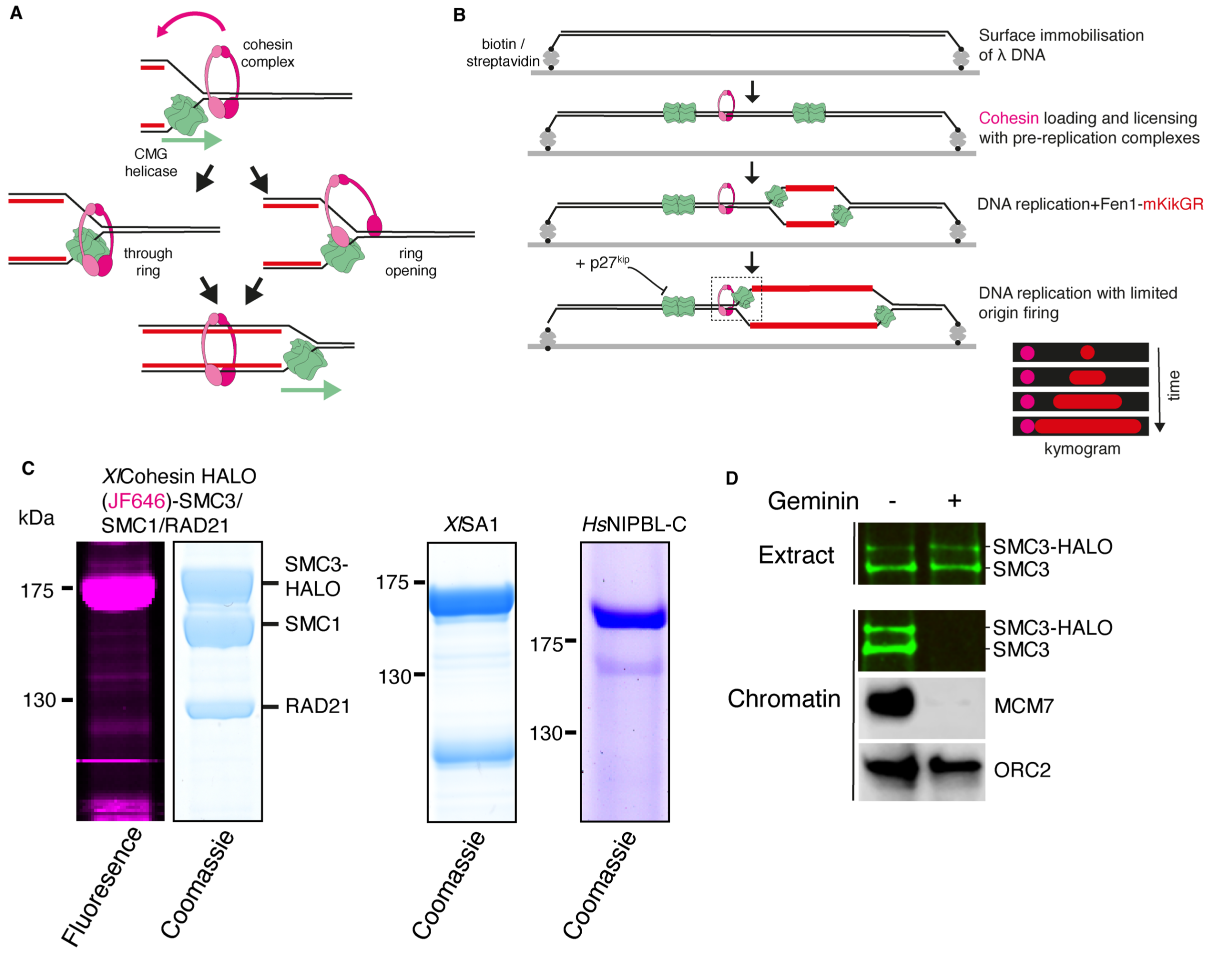

Fig. S1. Characterization of labeled Xenopus cohesin complexes. (**A**) Diagram showing the expected relocation of cohesin rings behind the replication fork, alongside possible models explaining how cohesin is transferred. (**B**) Schematic of single-molecule replication assay, where Fen1-mKikGR is used to visualize replication fork collision with pre-loaded cohesin. (**C**) Coomassie-stained SDS-PAGE gels showing *Xenopus laevis* cohesin trimer (SMC3-Halo(JF646)/SMC1/RAD21), *Xl*SA1 and *Hs*NIPBL^{c}. (**D**) Western blot showing Geminin sensitivity of JF646-cohesin loading onto chromatin in *Xenopus* egg extracts. SMC3 was blotted with a fluorescent secondary antibody, whilst MCM7 and ORC2 were probed with HRP-labeled secondary antibody and chemiluminescence. Loading of recombinant cohesin (SMC3-Halo) onto chromatin is comparable to that of endogenous cohesin (SMC3) in egg extracts.

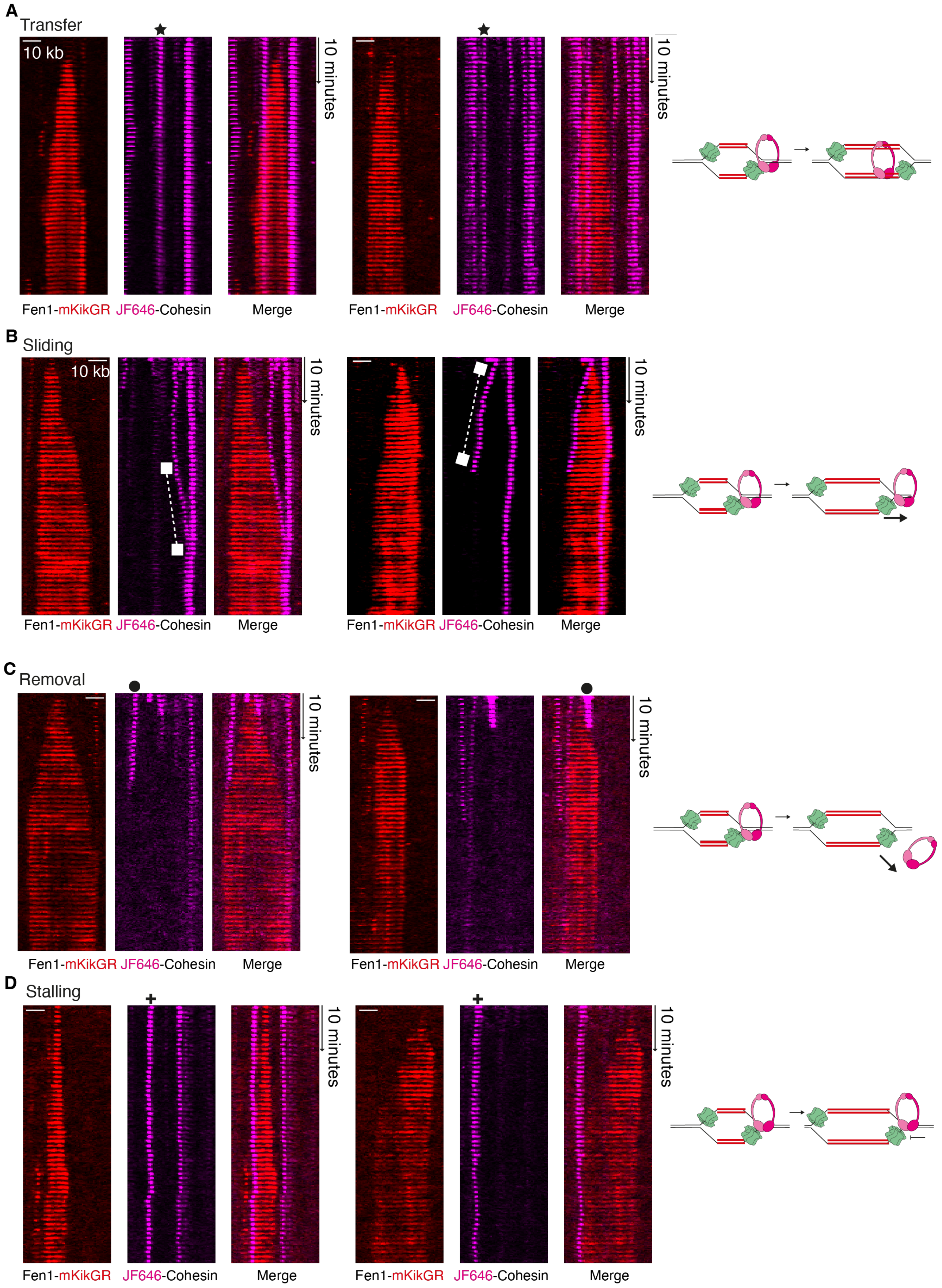
A
Transfer
10 kb
10 minutes
Fen1-mKikGR
JF646-Cohesin
Merge
B
Sliding
10 kb
10 minutes
Fen1-mKikGR
JF646-Cohesin
Merge
C
Removal
10 minutes
Fen1-mKikGR
JF646-Cohesin
Merge
D
Stalling
10 minutes
Fen1-mKikGR
JF646-Cohesin
Merge

Fig. S2. Different cohesin fates upon replication fork collision in *Xenopus* egg extracts. Kymograms showing real-time TIRF imaging of surface-tethered λ DNA during replication from single origins in *Xenopus* egg extracts. (**A**) In these examples JF646-cohesin (magenta) is incorporated into Fen1-mKikGR (red) labeled nascent DNA. Kymograms showing (**B**) cohesin sliding ahead of replication forks, (**C**) cohesin removal following replication fork arrival, and (**D**) replication fork stalling upon reaching cohesin.

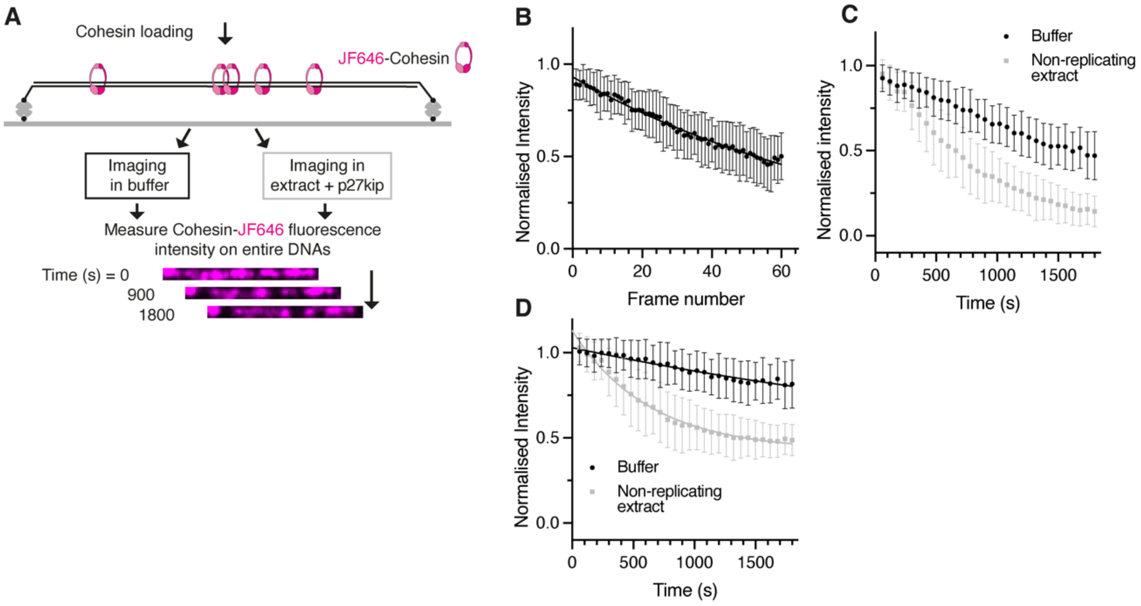

Fig. S3. Quantification of rate of cohesin loss from tethered DNAs. (**A**) Schematic showing experimental set-up for comparing disappearance of cohesin-associated fluorescence in buffer and in extract supplemented with p27^{kip} to prevent origin firing (non-replicating extracts). (**B**) Normalized fluorescence intensities for cohesin-JF646 loaded on DNA during continuous imaging in buffer (mean ± SD, n=50). Images were taken every 0.5 seconds (100 ms exposure) to minimize cohesin dissociation from DNA. (**C**) Normalized fluorescence intensities for cohesin-JF646 loaded on DNA in buffer or extract supplemented with p27^{kip} (mean ± SD, n=50 for each condition). Images were taken every 60 s for 1800 s (100 ms exposure). (**D**) Normalized fluorescence intensities as for (C) after correction for photobleaching. The fitted curve in (B) was re-calculated to estimate photobleaching during imaging with a 60 s lapse time. The difference between the starting intensity value (= 1.0) and an estimated intensity value after photobleaching was added to individual intensity values.

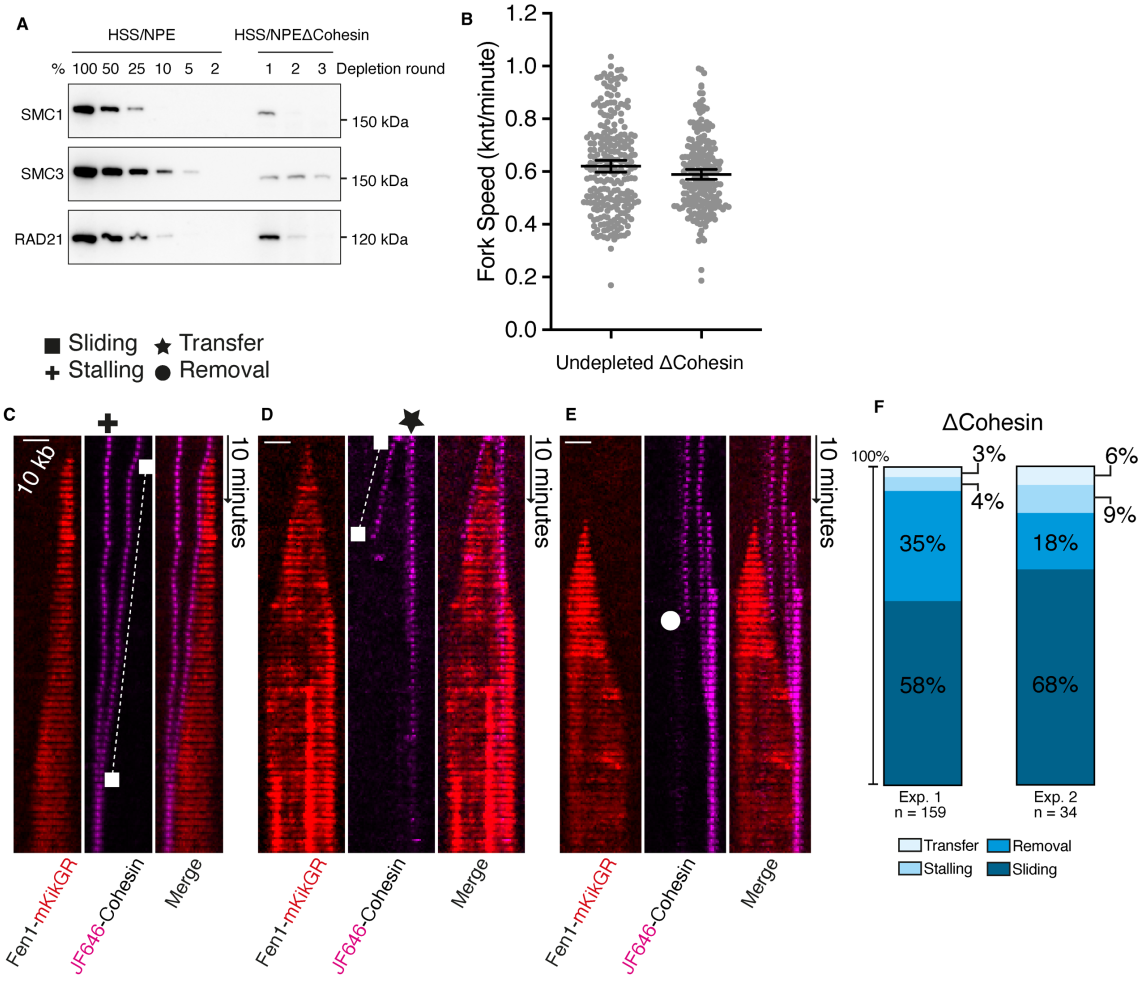

Fig. S4. Removal of free cohesin does not impact cohesin fate upon fork collision. (**A**) Western blot showing >95% removal of Smc1, Smc3 and Rad21 subunits from replication extract after immunodepletion. (**B**) Quantification of fork speeds with Fen1-mKikGR in undepleted (0.62 kb/minute, n=237) and cohesin-depleted (0.59 kb/minute, n=220) extracts. Mean shown with 95% CI. (**C**) to (**E**) Representative kymograms showing replication fork collision with pre-loaded cohesin in cohesin-depleted replication extracts. Origin firing is limited by addition of $p27^{kip}$. (**F**) Primary cohesin fate upon collision by replication forks in cohesin-depleted extracts. Two biological repeats are shown. Note that for Exp. 2, the data do not add up to 100% due to rounding.

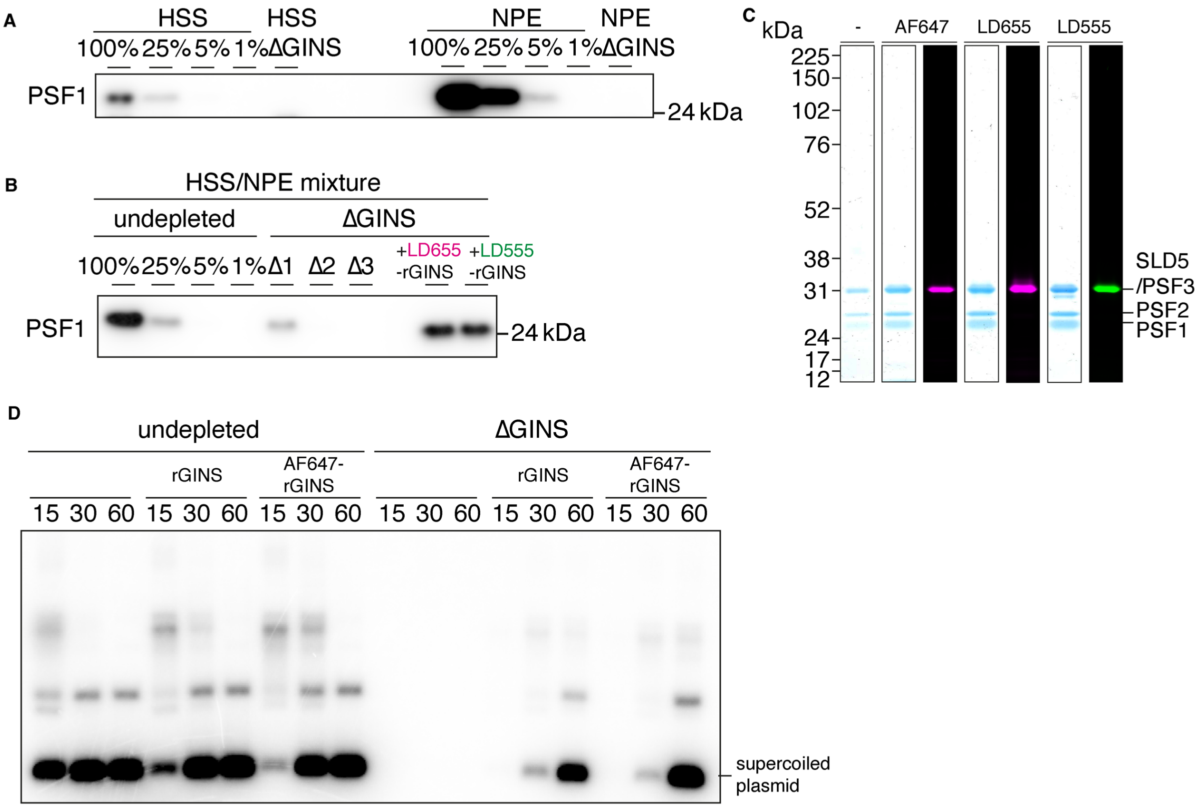

Fig. S5. Establishing DNA replication in *Xenopus* egg extracts with labeled GINS.
(**A**) Western blot showing GINS immunodepletion from HSS and NPE extracts separately (these extracts are used for bulk replication assays in (D)). (**B**) Western blot showing GINS immunodepletion from a mixture of HSS and NPE (these extracts are used for single-molecule replication assays). (**C**) Coomassie-stained (blue) or fluorescence-scanned SDS-PAGE gel showing unlabeled, AF647 / LD655- (magenta) and LD555- (green) labeled GINS complexes. (**D**) Plasmid DNA replication in undepleted and GINS-depleted extracts with addition of recombinant GINS complexes. Plasmid DNA containing ^{32}P-dATP was separated on a native agarose gel before visualization by autoradiography.

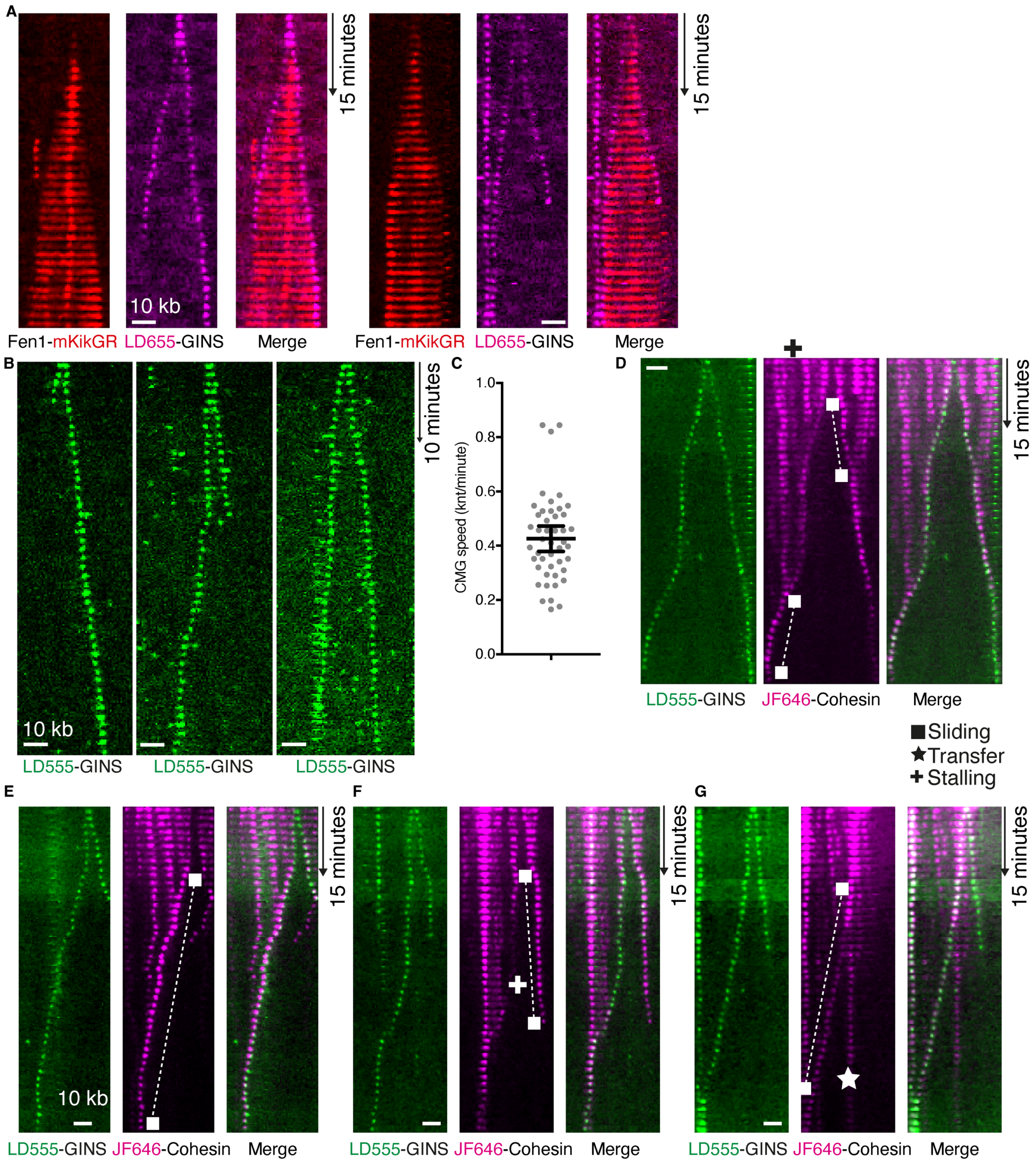

Fig. S6. DNA replication with individual labeled replisomes. (**A**) Kymograms showing limited origin firing with replisomes containing LD655-GINS (magenta) and nascent DNA labeled with Fen1-mKikGR (red). (**B**) Kymograms showing limited origin firing with replisomes containing LD555-GINS (green). (**C**) Replisome speeds with LD555-GINS (n=46, mean=0.426 kb/minute, shown with 95% CI). (**D** to **G**) Supplement to Fig. 2 with further examples of LD555-GINS / JF646-cohesin collisions after limited origin firing.

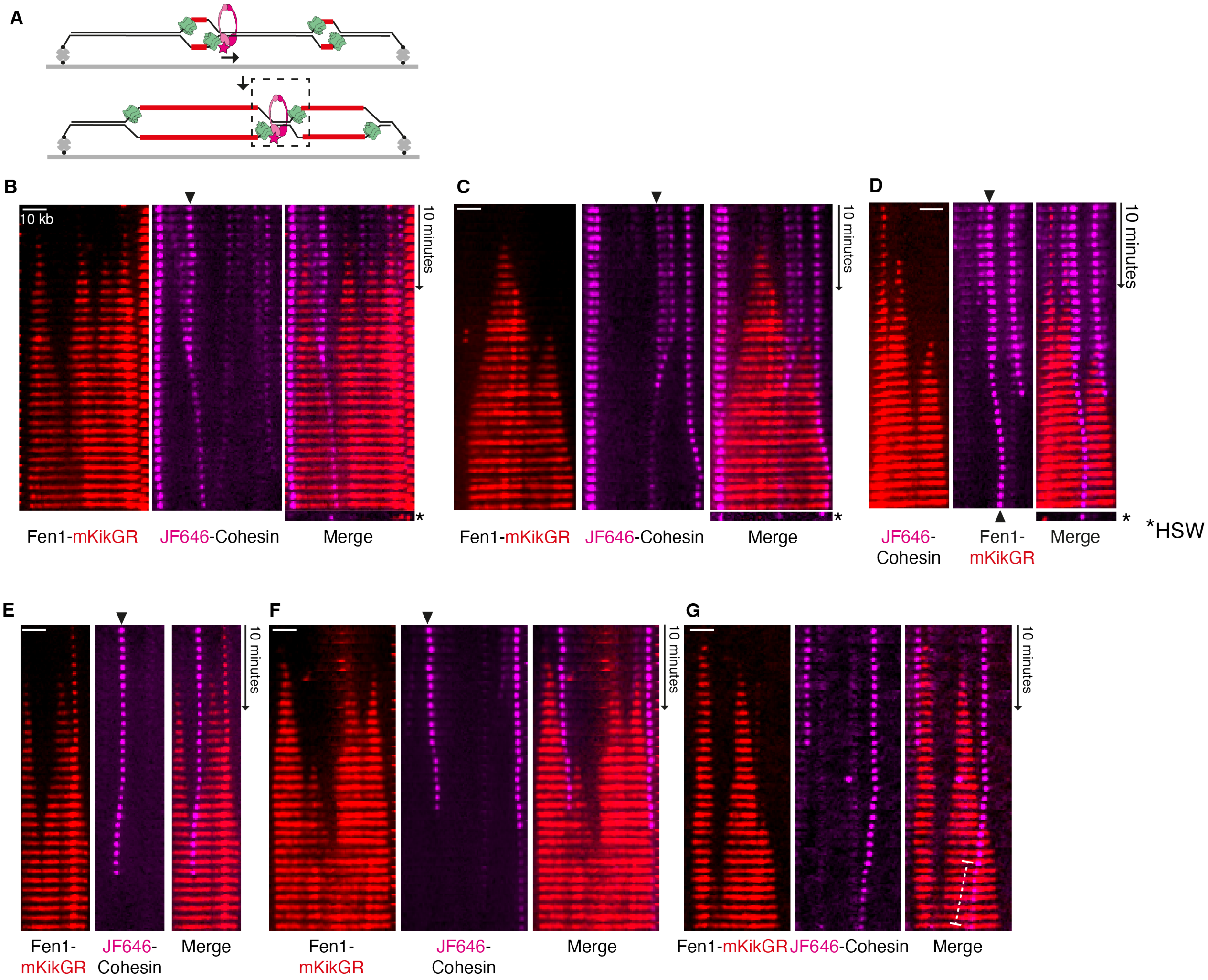

Fig. S7. Cohesin fates at positions where replication forks converge. (**A**) Schematic showing DNA replication from multiple origins on tethered DNAs. Nascent DNA is labeled with Fen1-mKikGR. Cohesin pushed ahead of a replication fork is visualized when meeting a converging replication fork. (**B** to **G**) Examples of JF646-cohesin fate at positions of converging Fen1-mKikGR labeled replication forks.

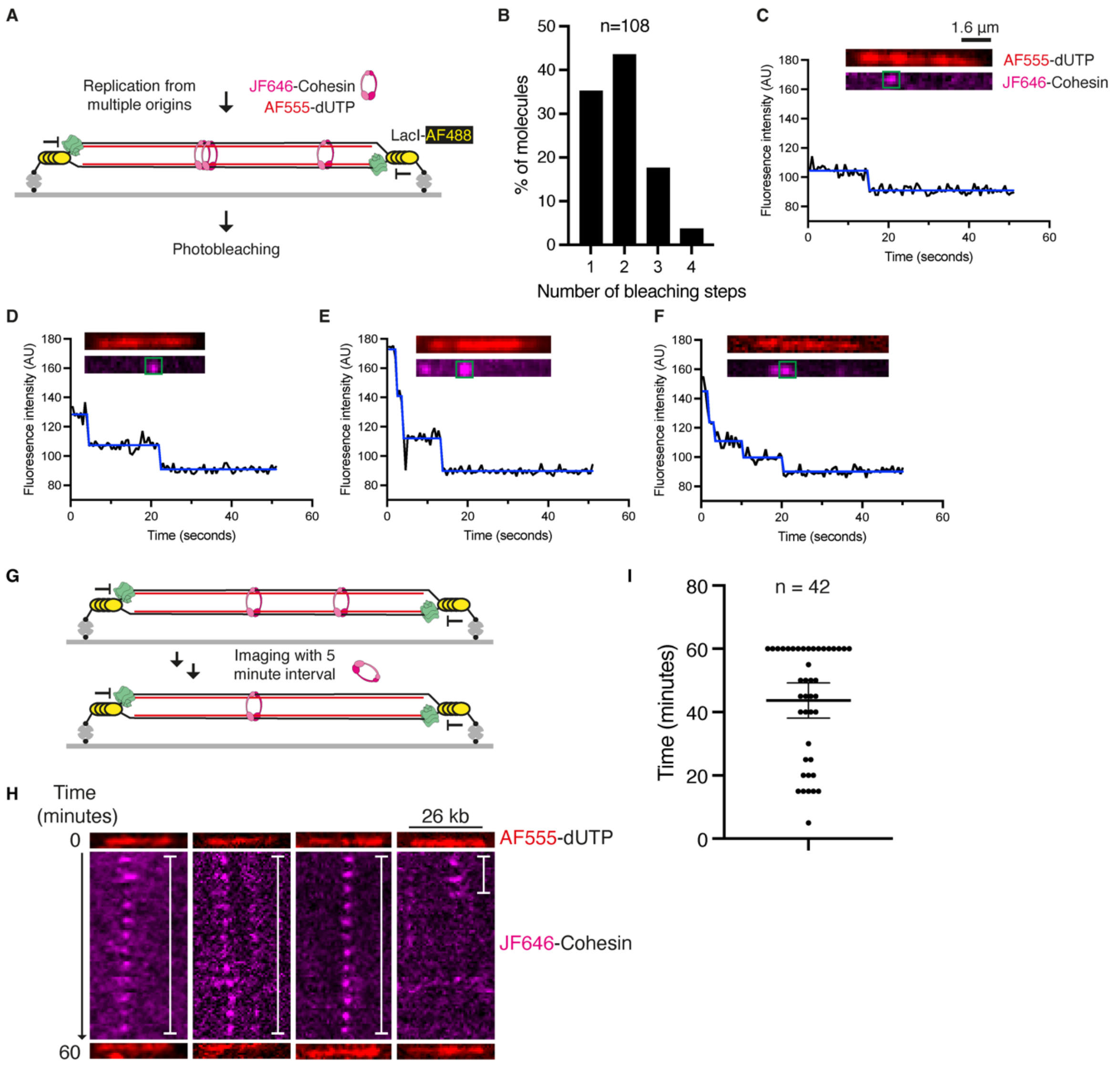

Fig. S8. Photobleaching analysis of cohesin after completion of DNA replication. (**A**) A tethered 26 kb DNA that is flanked by LacI-bound *lacO* repeats is loaded with JF646-cohesin complexes. DNAs are replicated from multiple origins in extract supplemented with AF555-dUTP to label nascent DNA, then washed with replication extract (to remove unincorporated AF555-dUTP) whilst maintaining LacI blockade of forks (see Fig. 3 for detailed explanation). (**B**) Histogram showing the number of photobleaching steps detected using AutoStepfinder in each spot (n=108, from two experiments). (**C**)-(**F**) Representative fluorescence intensity traces showing photobleaching of cohesin molecules (black line). These examples have 1, 2, 3, and 4 detected steps (blue line). (**G**) Experimental design for measuring cohesin stability after DNA replication termination. LacI arrays paused forks at the DNA ends after replication completion. AF555-labeled DNAs and JF646-cohesin were imaged with an increased lapse time (every 5 minutes for 60 minutes) to minimize photobleaching. Where cohesin was observed in the center of stretched DNAs after 60 minutes, the time cohesin remained was measured. (**H**) Kymograms showing cohesin retention on DNA after completion of DNA replication. Images of AF555-labeled DNA at the same positions are shown at the start and end of replication. (**I**) Cohesin retention time on DNAs after completion of DNA replication (43.7 ± 17.8, mean ± S.D.).

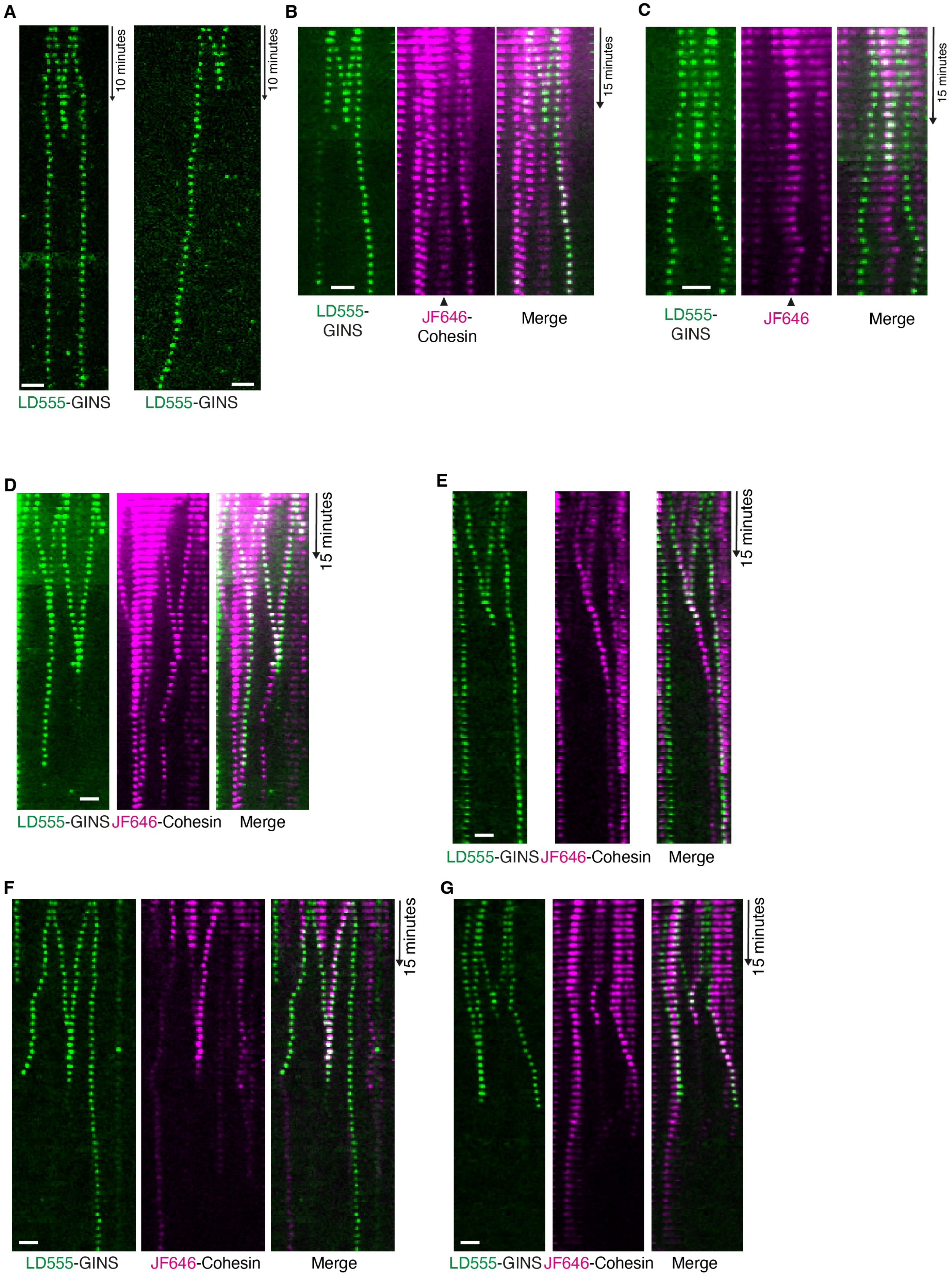
A
10 minutes
10 minutes
LD555-GINS
LD555-GINS
B
15 minutes
LD555-GINS
JF646-Cohesin
Merge
C
15 minutes
LD555-GINS
JF646
Merge
D
15 minutes
LD555-GINS
JF646-Cohesin
Merge
E
15 minutes
LD555-GINS
JF646-Cohesin
Merge
F
15 minutes
LD555-GINS
JF646-Cohesin
Merge
G
15 minutes
LD555-GINS
JF646-Cohesin
Merge

Fig. S9. Cohesin fates during DNA replication termination. (**A**) Kymograms showing DNA replication with LD555-GINS after firing from multiple origins. (**B** to **E**) Additional representative examples of replisomes (LD555-GINS) pushing JF646-cohesin to sites of replication termination. (B) and (C) are examples in which cohesin remains on DNA following replisome disassembly. In (D) and (E) cohesin remains then moves from the site of replisome disassembly. Cohesin moving after termination likely results from sister DNA collapsing from coverslip surface after one replisome reaches the tethered DNA end (see fig. S13 B and C). In (F) and (G) cohesin is removed during replisome disassembly.

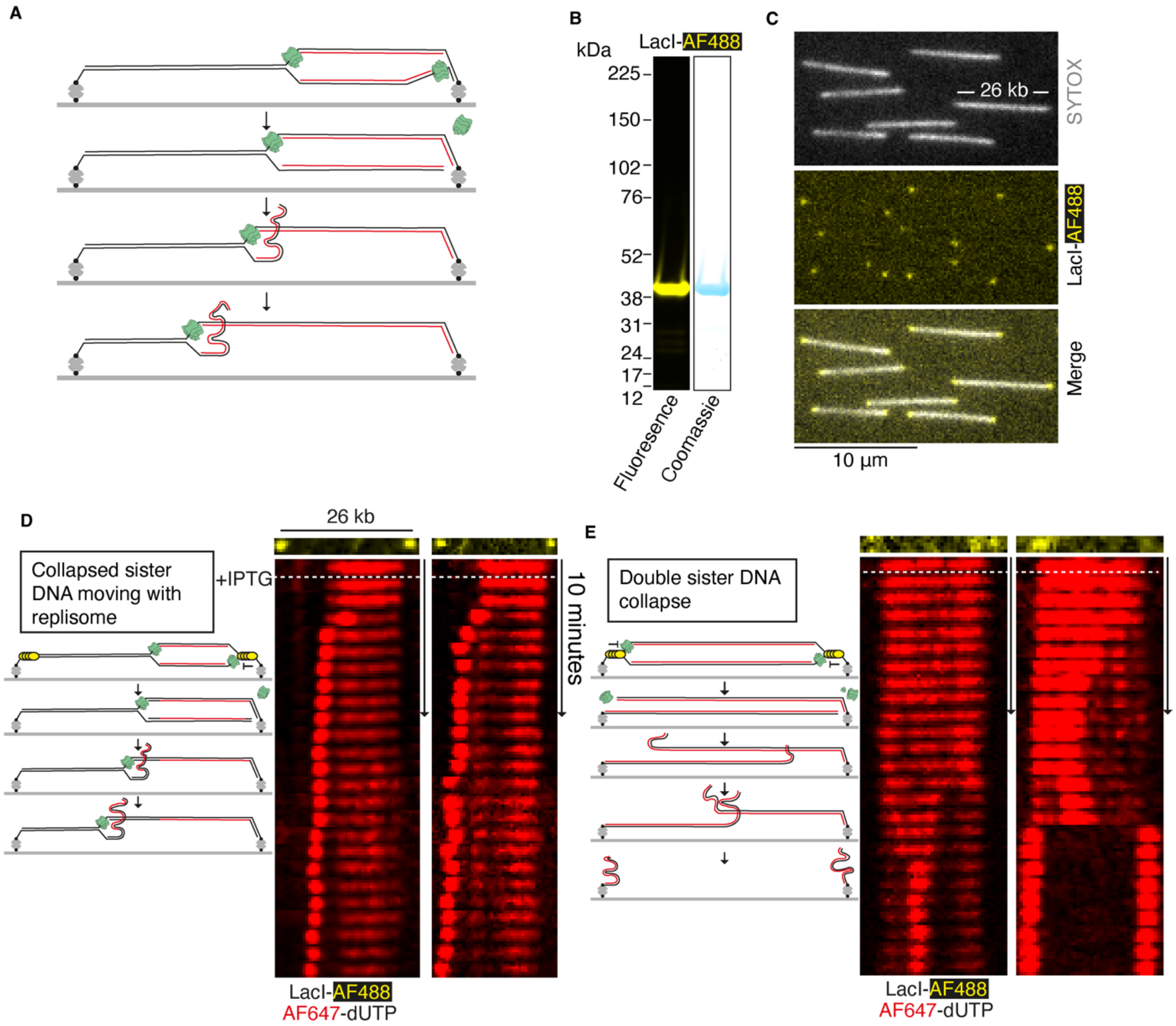

Fig. S10. DNA templates to study sister DNA collapse after replication. (**A**) Schematic showing one replisome reaching the DNA end and the sister DNA being liberated from surface-tethered DNA. The collapsed sister DNA localizes with the leftwards moving replisome. (**B**) SDS-PAGE gel showing purified LacI-AF488. (**C**) TIRF images of SYTOX Orange stained DNA bound by LacI-AF488. (**D**) Kymogram examples of newly replicated sister DNAs collapsing and colocalizing with the replisome. (**E**) Examples where both newly replicated sister DNAs collapse to the same position on surface-tethered DNA. In the right-hand example, the two new sister DNAs completely separate.

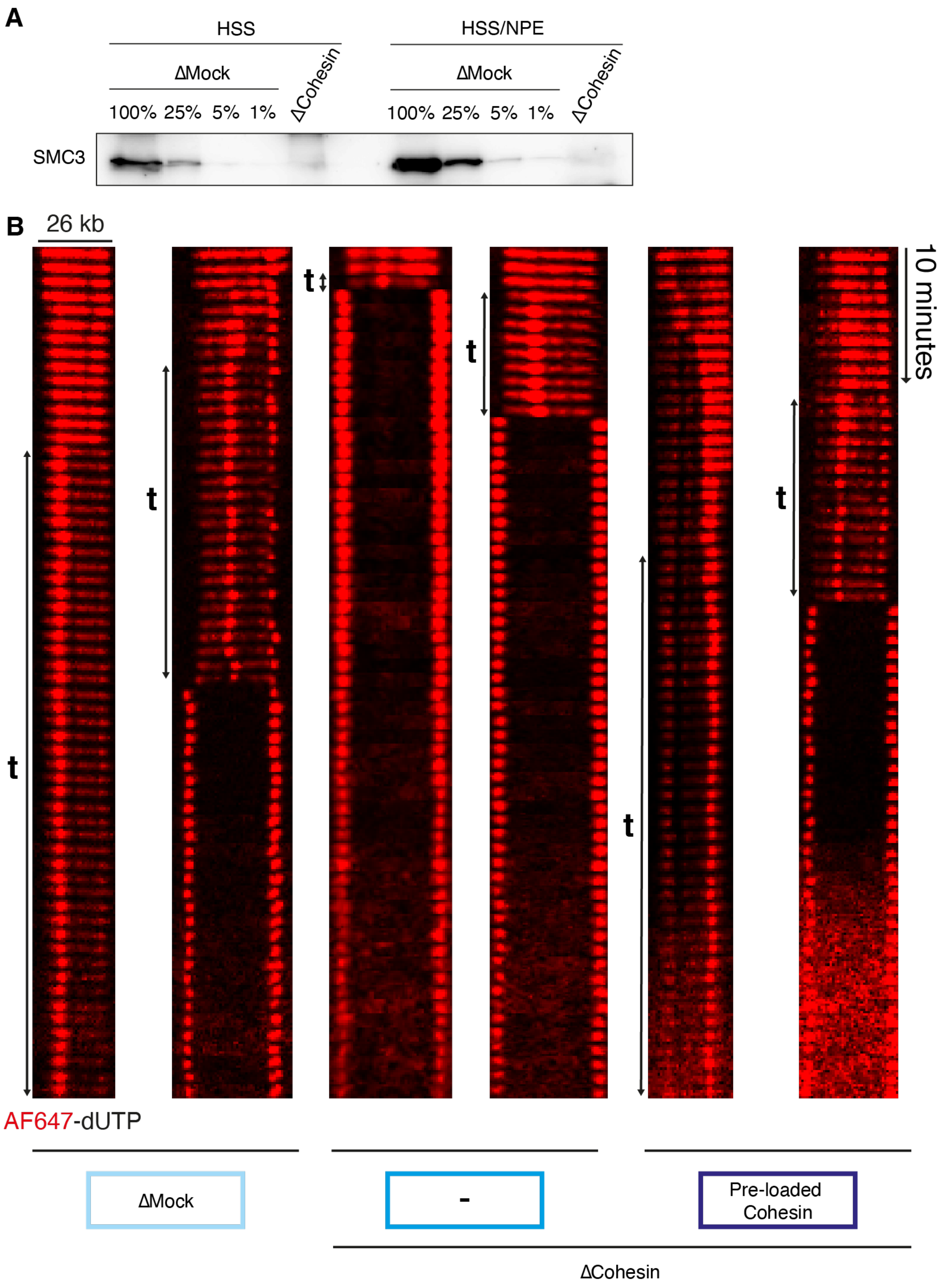

Fig. S11. Immunodepletion shows cohesin-dependent interaction tethering of replicated sister DNAs. (**A**) Western blot showing mock-depleted and cohesin-depleted extracts used for sister DNA collapse experiments. HSS was used for DNA licensing and HSS/NPE mixtures were used for replication. (**B**) Representative kymograms of double sister DNA collapse events where the time collapsed sister DNAs remain together is indicated. The conditions used for each example are indicated.

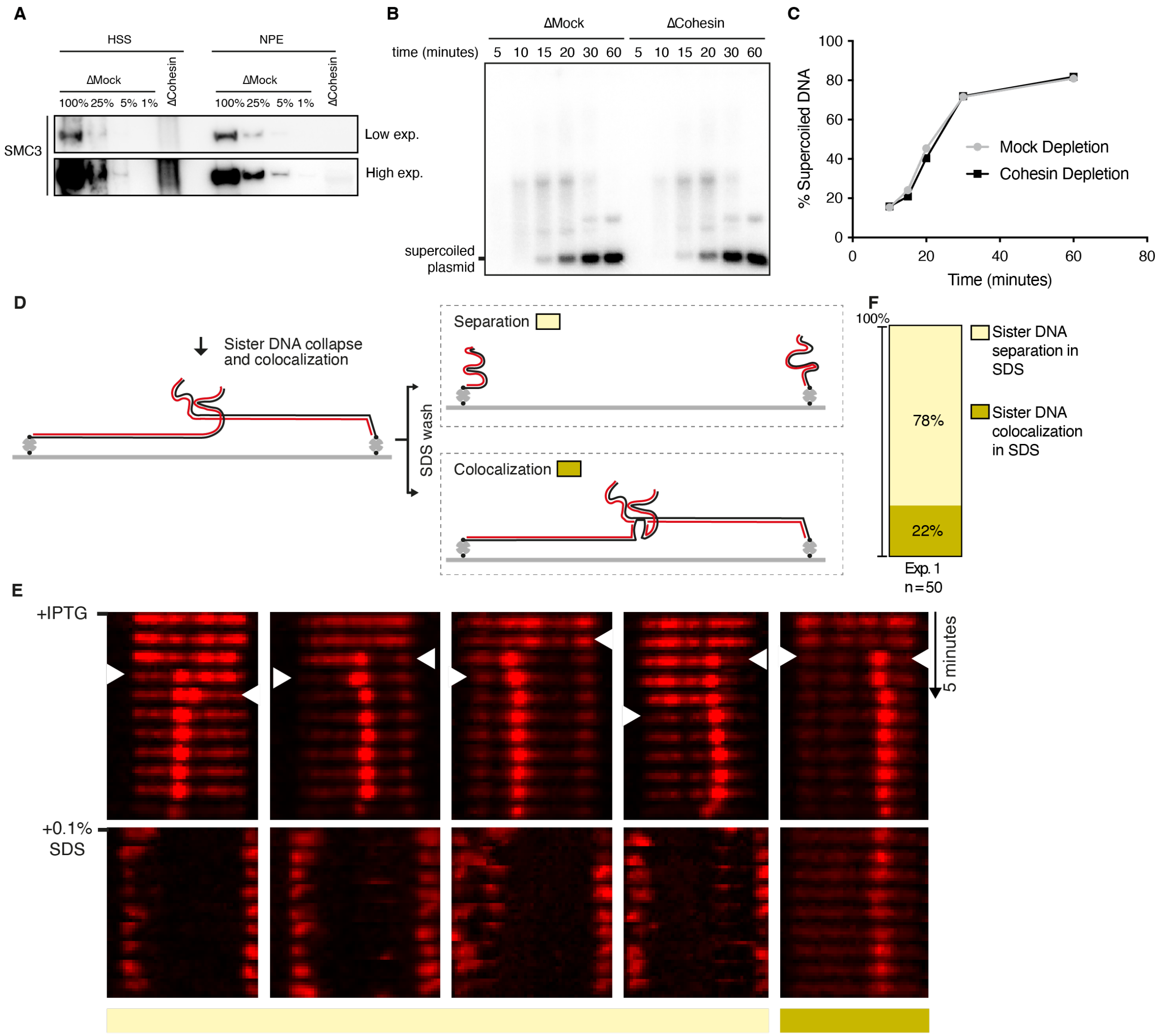

Fig. S12. Colocalization of sister DNAs is mediated by protein-DNA interactions,
(**A**) Western blot comparing mock-depleted and cohesin-depleted HSS and NPE extracts used for comparing bulk DNA replication timing. (**B**) Plasmid DNA replication in extracts. DNA containing ^{32}P-dATP is separated on a native agarose gel before visualization by autoradiography. (**C**) Quantification of supercoiled DNA shown in (B). The lack of difference in replication kinetics between the two conditions suggests cohesin does not impact completion of replication during fork convergence. (**D**) Schematic showing SDS-washing experiment after sister DNA collapse. After allowing sister DNA collapse to occur, flow cells are washed with buffer containing 0.1% SDS, which disrupts protein-DNA (but not DNA-DNA) interactions. For sister DNAs that colocalized before addition of SDS, instances of sister DNA separation and sister DNA colocalization are measured. (**E**) Kymogram examples showing sister DNA fates after washing with SDS-containing buffer. (**F**) Quantification of experiment shown in (D) and (E).

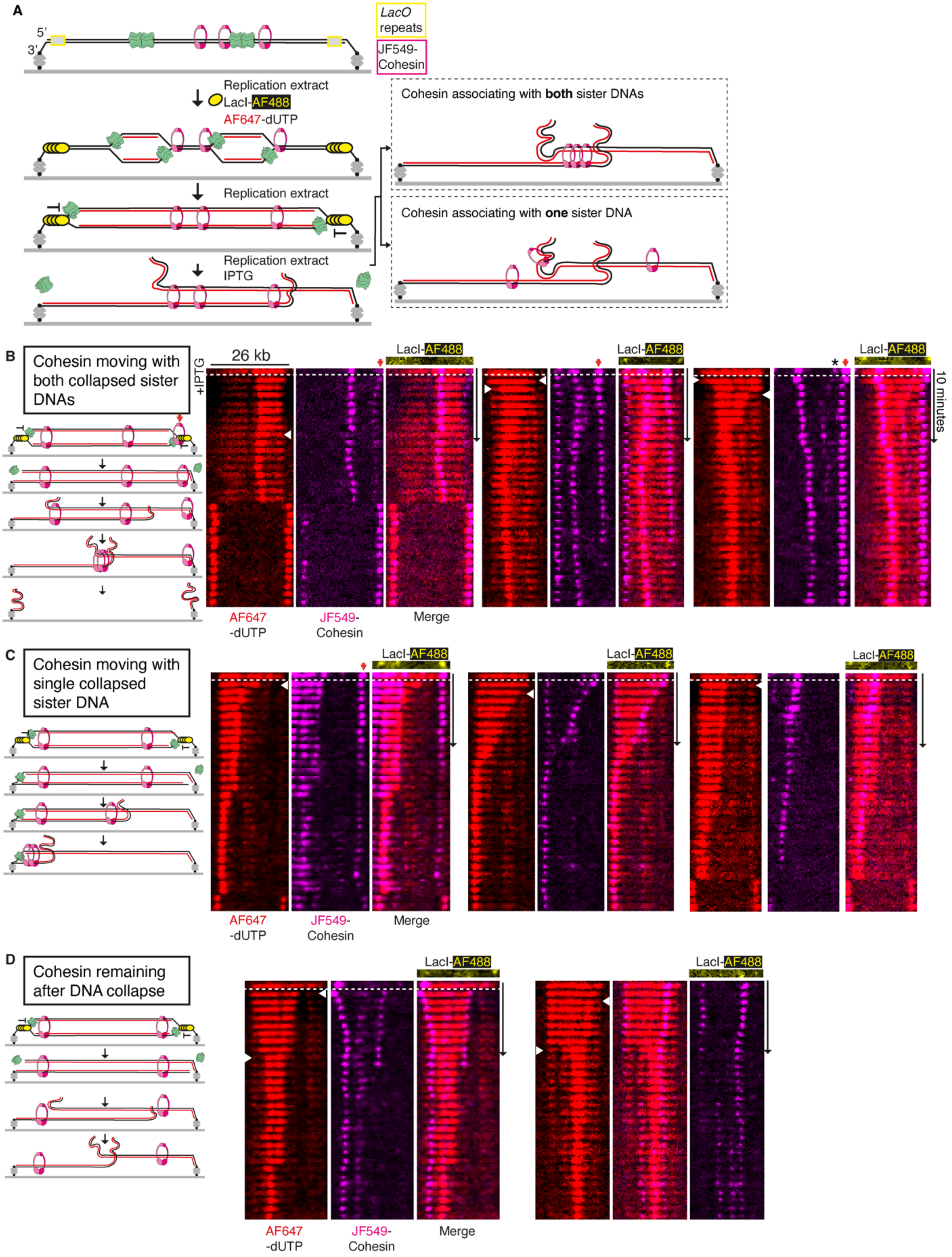
A
5'
3'
LacO repeats
JF549-Cohesin
Replication extract
LacI-AF488
AF647-dUTP
Replication extract
Replication extract
IPTG
Cohesin associating with both sister DNAs
Cohesin associating with one sister DNA
B
Cohesin moving with both collapsed sister DNAs
+IPTG
26 kb
LacI-AF488
10 minutes
AF647 -dUTP
JF549- Cohesin
Merge
C
Cohesin moving with single collapsed sister DNA
LacI-AF488
AF647 -dUTP
JF549- Cohesin
Merge
D
Cohesin remaining after DNA collapse
LacI-AF488
AF647 -dUTP
JF549- Cohesin
Merge

Fig. S13. Cohesin fates after sister DNA collapse. (**A**) Diagram showing the assay used to investigate whether cohesin binds one or both sister DNAs after collapse. (**B**) Kymogram examples showing cohesin moving with both collapsed sister DNAs, as seen in Fig. 3E. *marks a cohesin that does not remain with collapsing strands. The red arrow indicates cohesin at the end of DNA tethers, which is excluded from the analysis. (**C**) Kymogram examples where cohesin moves with a single collapsed sister DNA. (**D**) Kymogram examples where cohesin remains on surface tethered DNA after sister DNAs collapse.

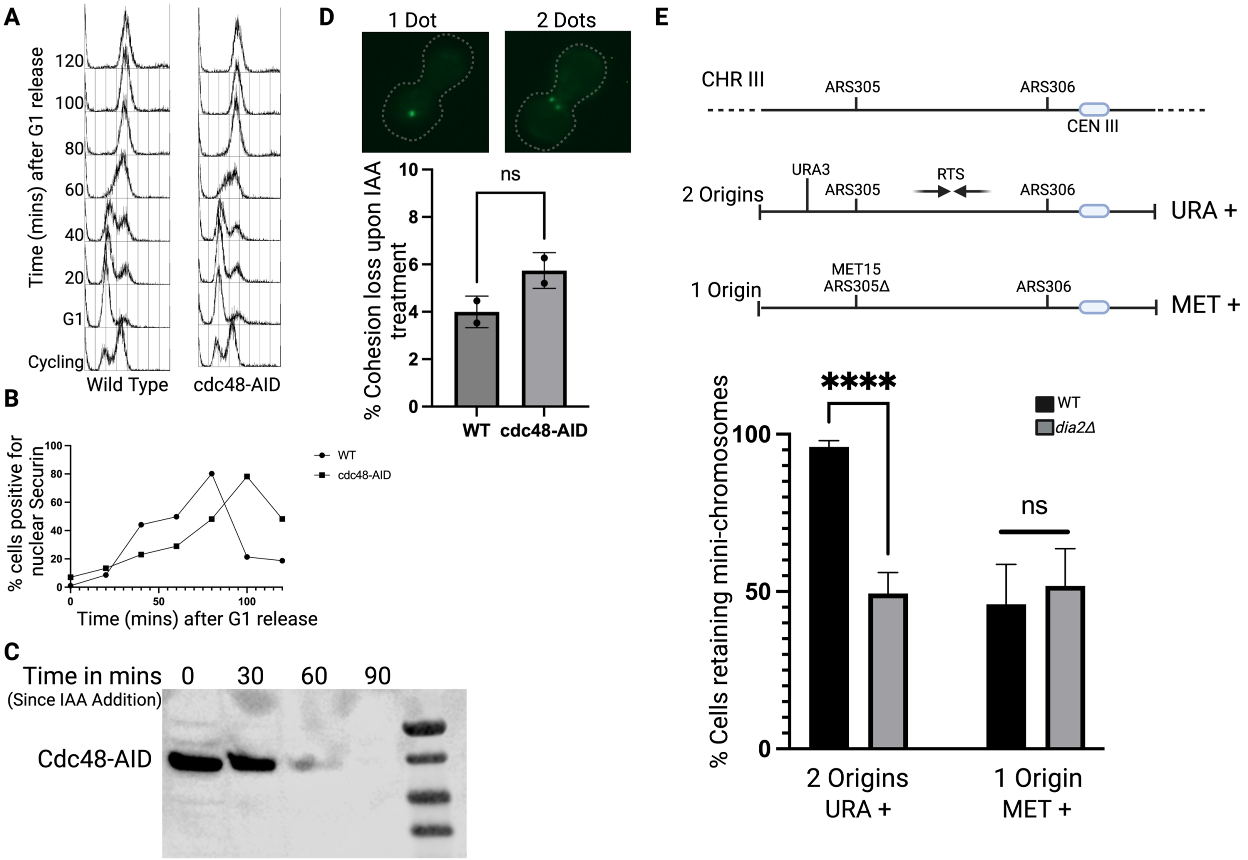

Fig. S14. Replication and cell cycle progression of cdc48-AID strain. (**A**) Wild type and cdc48-AID cells were synchronized in G1 and released by filtration into YPD containing 10 µg/mL nocodazole and 5 µM IAA. Samples were drawn at indicated times and the cells were fixed in 50% ethanol and the DNA content of the cells measured by FACS. (**B**) Wild type and cdc48-AID cells were synchronized in G1 and released by filtration into YPD supplemented with 5 µM IAA. Samples were drawn at indicated times and the cells were fixed in 50% ethanol. The cells were subjected to in situ immunofluorescence to count the number of cells displaying nuclear PDS1. Cdc48 depletion did not compromise replication completion and induced a minor delay in anaphase entry in yeast cells. (**C**) cdc48-AID cells were arrested in G1 and 5 µM IAA was added. Samples were drawn at the indicated times and cell lysate prepared and subjected to SDS-PAGE and western blot to detect Cdc48-AID. **(D)** Wild type and cdc48-AID cells were synchronized in G2 phase by addition of nocodazole (20 µg/ml). Samples were taken before and 90 minutes after addition of 5mM IAA. Cells were fixed with 4% formaldehyde for 60 minutes, washed with PBS and embedded into agar plugs. The cells were imaged as described in methods and scored for one or two GFP dots . The data shown (mean ± SD) are from 2 independent experiments and at least 100 cells were scored for each experimental condition. **(E)** Schematic representation of the strain harboring two short linear mini-chromosomes. The approximate position of the replication termination site (RTS) between the two high efficiency origins is shown. Wild type and dia2 mutants were grown as described in methods to score for the % cells harboring the URA+ and MET+ mini-chromosomes. The data shown (mean ± SD) are from three independent experiments.

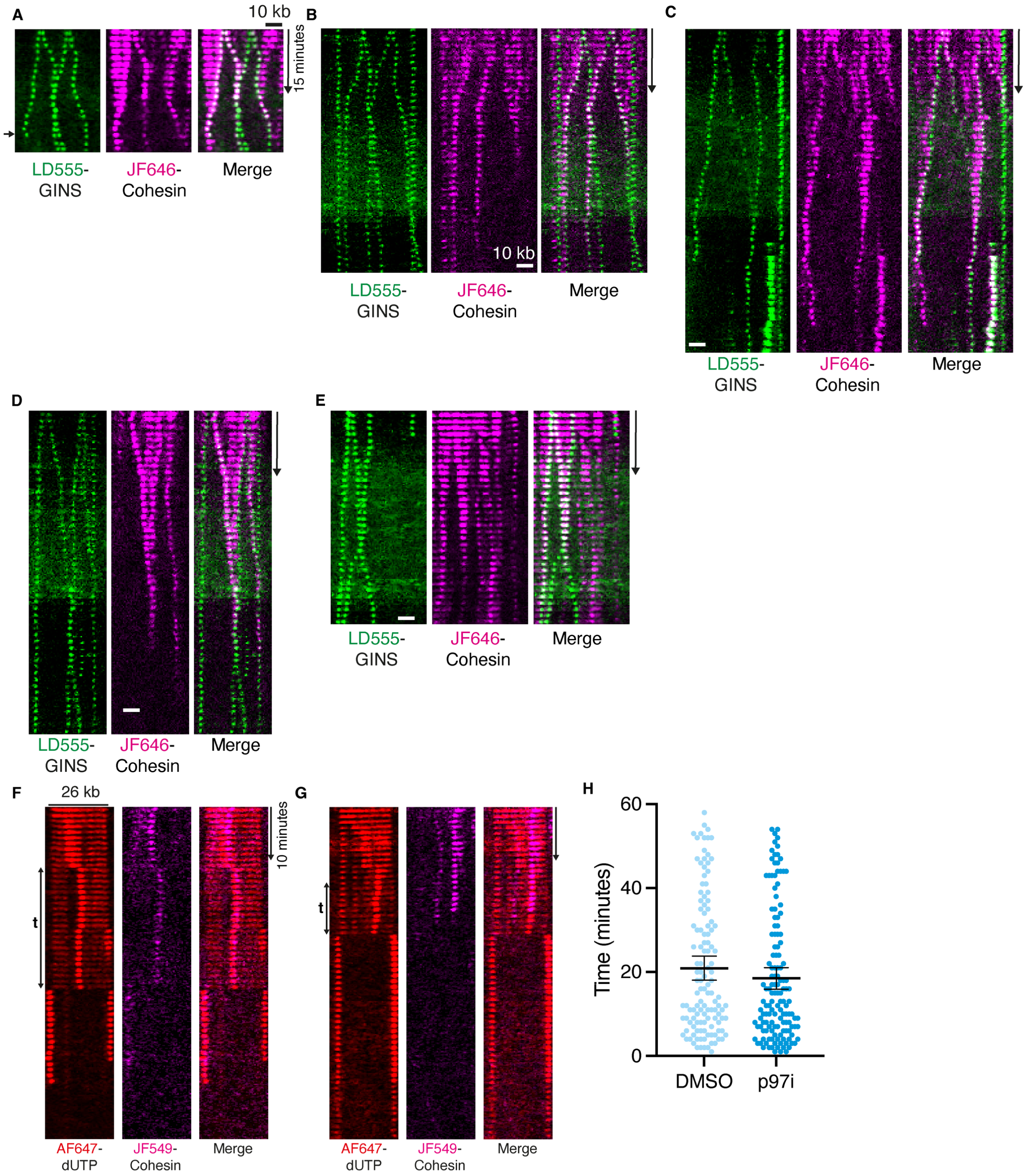

Fig. S15. Effect of inhibiting replisome disassembly on cohesin fate during DNA replication termination, and on sister chromatid cohesion. Example kymograms where replisome disassembly is inhibited and cohesin is visualized during replisome convergence. In examples (**A**) and (**B**), converging replisomes bypass one another and one replisome continues pushing a labeled cohesin. In example (**C**) converging replisomes stall during replication termination, which is coincident with a labeled cohesin. In (**D**) cohesin is removed when replisomes bypass one another, whilst in (**E**) cohesin remains at the position where converging replisomes bypass

one another. Example kymograms of sister strand collapse in extracts supplemented with (**F**) DMSO, or (**G**) p97i (NMS-873). Fluorescent cohesin was pre-loaded on tethered DNAs for these experiments. The time that sister DNAs colocalize is indicated. (**H**) Plots showing timings of sister chromatid colocalization ±p97i. Data were collected from two experiments for each condition (DMSO, n = 124 and p97i, n = 140).

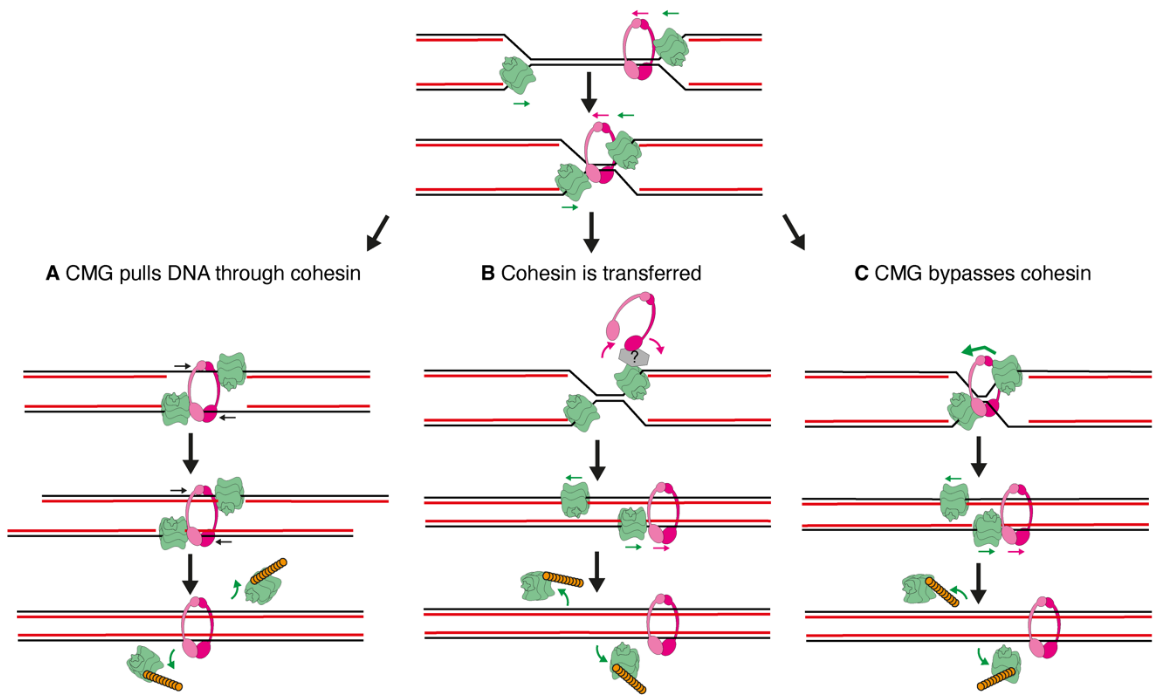

Fig. S16. Models for cohesion establishment during replication termination. Cohesin is pushed to replication termination sites by the replisome. (**A**) As replisomes converge, they are on either side of cohesin at termination sites. The final stretch of DNA inside the cohesin ring is melted, allowing replisomes to pull unreplicated DNA through cohesin rings. As replisomes lose interaction with the replication fork, and move onto dsDNA, they are ubiquitylated and disassembled, whilst cohesin entraps sister DNAs. (**B**) During replication termination, replisome-associated factors transfer cohesin behind the replication fork. This process involves transient opening of the cohesin ring, with cohesin entrapping new sister DNAs. This could involve a mechanism where cohesin sequentially captures dsDNA and ssDNA (*42*). (**C**) During replication termination with cohesin present, one replisome bypasses the cohesin ring. This might involve a mechanism similar to DNA-protein crosslink bypass (*24*), or the replisome passaging through the cohesin ring. Rearrangement of either cohesin or replisome structure might facilitate passage of the replication fork through the cohesin ring. We note the possibility of the model described in (A) operating under conditions where DNA is not constrained with models B or C taking place when DNA is under tension as in our experiments, which would allow CMG to keep pushing cohesin after fork convergence as seen in Fig. 4B.

Table S1. Yeast strains used in this study

Strain number	Genotype
**7480**	MAT a, ura3::URA3 tetOs, leu2::LEU2 tetR-GFP, Pds1myc18::TRP, ADE
**29208**	MAT a, ura3::URA3 tetOs, leu2::LEU2 tetR-GFP, Pds1myc18::TRP,dia2::HIS3
**29265**	MAT a, ura3::URA3 tetOs, leu2::LEU2 tetR-GFP, Pds1myc18::TRP, ADE
**29345**	MAT a, his3::ADH1 promoter-OsTIR1-9myc::HIS3, cdc48-AID::KAN MX ura3::URA3 tetOs, leu2::LEU2 tetR-GFP, Pds1myc18::TRP(K.Lactis)
**29393**	MAT alpha, ura3::URA3 tetOs, leu2::LEU2 tetR-GFP, ADE2, dia2-13A::URA3
**29523**	MATa, ade2-1, trp1-1, can1-100, leu2-3,112,his3-11,15, ura3, GAL, psi+ ade3::hphMX met15::KAN MX<br>Trunc Chr i TEL HIS3 loxP-CHRIII seq31081 ARS305, ADE3 URA3 loxP integ at 43614 (at YCL048w) ARS306 CHRIII seq 96070 integrated CEN4 TRP1 TEL<br><br>Trunc Chr ii TEL HIS3 loxP-CHRIII seq31081 ARS305::MET15 ARS306 CHRIII seq 96070 integrated CEN4 TRP1 TEL
**29525**	MATa, ade2-1, trp1-1, can1-100, leu2-3,112,his3-11,15, ura3, GAL, psi+ ade3::hphMX met15::KAN MX<br>Trunc Chr i TEL HIS3 loxP-CHRIII seq31081 ARS305, ADE3 URA3 loxP integ at 43614 (at YCL048w) ARS306 CHRIII seq 96070 integrated CEN4 TRP1 TEL<br>dia2::Leu2<br>Trunc Chr ii TEL HIS3 loxP-CHRIII seq31081 ARS305::MET15 ARS306 CHRIII seq 96070 integrated CEN4 TRP1 TEL

Supplementary Movies

Movie S1. Sliding of JF646-cohesin (magenta) ahead of the replication fork. Nascent DNA labeled with Fen1-mKiKGR (red). Related to Fig. 1B.

Movie S2. LD555-labeled CMG pushing JF646-cohesin during fork progression. Related to Fig. 1D.

Movie S3. Cohesin relocalization to a DNA replication termination site. Related to Fig. 2A.

Movie S4. Visualization of CMG disassembly and cohesin retention at a replication termination site. Related to fig. S9B.

Movie S5. Collapse and separation of sister DNAs upon completion of DNA replication. Related to Fig. 3B.

Movie S6. JF549-cohesin associating with collapsed sister DNAs. Related to Fig. 3D.

Movie S7. Converging replisomes bypassing one another with one replisome continuing to push a labeled cohesin. Related to Fig. 4B.

References and Notes

1. S. Yatskevich, J. Rhodes, K. Nasmyth, Organization of chromosomal DNA by SMC complexes. *Annu. Rev. Genet.* **53**, 445–482 (2019). doi:10.1146/annurev-genet-112618-043633 Medline

2. I. F. Davidson, B. Bauer, D. Goetz, W. Tang, G. Wutz, J. M. Peters, DNA loop extrusion by human cohesin. *Science* **366**, 1338–1345 (2019). doi:10.1126/science.aaz3418 Medline

3. Y. Kim, Z. Shi, H. Zhang, I. J. Finkelstein, H. Yu, Human cohesin compacts DNA by loop extrusion. *Science* **366**, 1345–1349 (2019). doi:10.1126/science.aaz4475 Medline

4. R. Ciosk, M. Shirayama, A. Shevchenko, T. Tanaka, A. Toth, A. Shevchenko, K. Nasmyth, Cohesin's binding to chromosomes depends on a separate complex consisting of Scc2 and Scc4 proteins. *Mol. Cell* **5**, 243–254 (2000). doi:10.1016/S1097-2765(00)80420-7 Medline

5. T. S. Takahashi, P. Yiu, M. F. Chou, S. Gygi, J. C. Walter, Recruitment of *Xenopus* Scc2 and cohesin to chromatin requires the pre-replication complex. *Nat. Cell Biol.* **6**, 991–996 (2004). doi:10.1038/ncb1177 Medline

6. G. Zheng, M. Kanchwala, C. Xing, H. Yu, MCM2-7-dependent cohesin loading during S phase promotes sister-chromatid cohesion. *eLife* **7**, e33920 (2018). doi:10.7554/eLife.33920 Medline

7. S. P. Bell, K. Labib, Chromosome duplication in *Saccharomyces cerevisiae*. *Genetics* **203**, 1027–1067 (2016). doi:10.1534/genetics.115.186452 Medline

8. J. T. P. Yeeles, A. Janska, A. Early, J. F. X. Diffley, How the eukaryotic replisome achieves rapid and efficient DNA replication. *Mol. Cell* **65**, 105–116 (2017). doi:10.1016/j.molcel.2016.11.017 Medline

9. A. Gambus, R. C. Jones, A. Sanchez-Diaz, M. Kanemaki, F. van Deursen, R. D. Edmondson, K. Labib, GINS maintains association of Cdc45 with MCM in replisome progression complexes at eukaryotic DNA replication forks. *Nat. Cell Biol.* **8**, 358–366 (2006). doi:10.1038/ncb1382 Medline

10. M. Srinivasan, J. C. Scheinost, N. J. Petela, T. G. Gligoris, M. Wissler, S. Ogushi, J. E. Collier, M. Voulgaris, A. Kurze, K. L. Chan, B. Hu, V. Costanzo, K. A. Nasmyth, The cohesin ring uses its hinge to organize DNA using non-topological as well as topological mechanisms. *Cell* **173**, 1508–1519.e18 (2018). doi:10.1016/j.cell.2018.04.015 Medline

11. F. Uhlmann, K. Nasmyth, Cohesion between sister chromatids must be established during DNA replication. *Curr. Biol.* **8**, 1095–1101 (1998). doi:10.1016/S0960-9822(98)70463-4 Medline

12. H. Xu, C. Boone, G. W. Brown, Genetic dissection of parallel sister-chromatid cohesion pathways. *Genetics* **176**, 1417–1429 (2007). doi:10.1534/genetics.107.072876 Medline

13. M. Srinivasan, M. Fumasoni, N. J. Petela, A. Murray, K. A. Nasmyth, Cohesion is established during DNA replication utilising chromosome associated cohesin rings as well as those loaded de novo onto nascent DNAs. *eLife* **9**, e56611 (2020). doi:10.7554/eLife.56611 Medline

14. A. Lengronne, Y. Katou, S. Mori, S. Yokobayashi, G. P. Kelly, T. Itoh, Y. Watanabe, K. Shirahige, F. Uhlmann, Cohesin relocation from sites of chromosomal loading to places of convergent transcription. *Nature* **430**, 573–578 (2004). doi:10.1038/nature02742 Medline

15. B. Hu, N. Petela, A. Kurze, K.-L. Chan, C. Chapard, K. Nasmyth, Biological chromodynamics: a general method for measuring protein occupancy across the genome by calibrating ChIP-seq. *Nucleic Acids Res.* **43**, e132 (2015). doi:10.1093/nar/gkv670 Medline

16. J. Walter, L. Sun, J. Newport, Regulated chromosomal DNA replication in the absence of a nucleus. *Mol. Cell* **1**, 519–529 (1998). doi:10.1016/S1097-2765(00)80052-0 Medline

17. A. B. Loveland, S. Habuchi, J. C. Walter, A. M. van Oijen, A general approach to break the concentration barrier in single-molecule imaging. *Nat. Methods* **9**, 987–992 (2012). doi:10.1038/nmeth.2174 Medline

18. H. Yardimci, A. B. Loveland, A. M. van Oijen, J. C. Walter, Single-molecule analysis of DNA replication in *Xenopus* egg extracts. *Methods* **57**, 179–186 (2012). doi:10.1016/j.ymeth.2012.03.033 Medline

19. W. S. Hoogenboom, D. Klein Douwel, P. Knipscheer, *Xenopus* egg extract: A powerful tool to study genome maintenance mechanisms. *Dev. Biol.* **428**, 300–309 (2017). doi:10.1016/j.ydbio.2017.03.033 Medline

20. A. Losada, M. Hirano, T. Hirano, Identification of *Xenopus* SMC protein complexes required for sister chromatid cohesion. *Genes Dev.* **12**, 1986–1997 (1998). doi:10.1101/gad.12.13.1986 Medline

21. H. Yardimci, A. B. Loveland, S. Habuchi, A. M. van Oijen, J. C. Walter, Uncoupling of sister replisomes during eukaryotic DNA replication. *Mol. Cell* **40**, 834–840 (2010). doi:10.1016/j.molcel.2010.11.027 Medline

22. D. T. Gruszka, S. Xie, H. Kimura, H. Yardimci, Single-molecule imaging reveals control of parental histone recycling by free histones during DNA replication. *Sci. Adv.* **6**, eabc0330 (2020). doi:10.1126/sciadv.abc0330 Medline

23. H. W. Liu, C. Bouchoux, M. Panarotto, Y. Kakui, H. Patel, F. Uhlmann, Division of labor between PCNA loaders in DNA replication and sister chromatid cohesion establishment. *Mol. Cell* **78**, 725–738.e4 (2020). doi:10.1016/j.molcel.2020.03.017 Medline

24. J. L. Sparks, G. Chistol, A. O. Gao, M. Räschle, N. B. Larsen, M. Mann, J. P. Duxin, J. C. Walter, The CMG helicase bypasses DNA-protein cross-links to facilitate their repair. *Cell* **176**, 167–181.e21 (2019). doi:10.1016/j.cell.2018.10.053 Medline

25. K. B. Vrtis, J. M. Dewar, G. Chistol, R. A. Wu, T. G. W. Graham, J. C. Walter, Single-strand DNA breaks cause replisome disassembly. *Mol. Cell* **81**, 1309–1318.e6 (2021). doi:10.1016/j.molcel.2020.12.039 Medline

26. E. Low, G. Chistol, M. S. Zaher, O. V. Kochenova, J. C. Walter, The DNA replication fork suppresses CMG unloading from chromatin before termination. *Genes Dev.* **34**, 1534–1545 (2020). doi:10.1101/gad.339739.120 Medline

27. M. Minamino, T. L. Higashi, C. Bouchoux, F. Uhlmann, Topological in vitro loading of the budding yeast cohesin ring onto DNA. *Life Sci. Alliance* **1**, e201800143 (2018). doi:10.26508/lsa.201800143 Medline

28. J. M. Dewar, J. C. Walter, Mechanisms of DNA replication termination. *Nat. Rev. Mol. Cell Biol.* **18**, 507–516 (2017). doi:10.1038/nrm.2017.42 Medline

29. M. Maric, T. Maculins, G. De Piccoli, K. Labib, Cdc48 and a ubiquitin ligase drive disassembly of the CMG helicase at the end of DNA replication. *Science* **346**, 1253596 (2014). doi:10.1126/science.1253596 Medline

30. J. M. Dewar, E. Low, M. Mann, M. Räschle, J. C. Walter, CRL2^{Lrr1} promotes unloading of the vertebrate replisome from chromatin during replication termination. *Genes Dev.* **31**, 275–290 (2017). doi:10.1101/gad.291799.116 Medline

31. M. Jenkyn-Bedford, M. L. Jones, Y. Baris, K. P. M. Labib, G. Cannone, J. T. P. Yeeles, T. D. Deegan, A conserved mechanism for regulating replisome disassembly in eukaryotes. *Nature* **600**, 743–747 (2021). doi:10.1038/s41586-021-04145-3 Medline

32. J. Stigler, G. Ö. Çamdere, D. E. Koshland, E. C. Greene, Single-molecule imaging reveals a collapsed conformational state for DNA-bound cohesin. *Cell Rep.* **15**, 988–998 (2016). doi:10.1016/j.celrep.2016.04.003 Medline

33. I. F. Davidson, D. Goetz, M. P. Zaczek, M. I. Molodtsov, P. J. Huis In ’t Veld, F. Weissmann, G. Litos, D. A. Cisneros, M. Ocampo-Hafalla, R. Ladurner, F. Uhlmann, A. Vaziri, J. M. Peters, Rapid movement and transcriptional re-localization of human cohesin on DNA. *EMBO J.* **35**, 2671–2685 (2016). doi:10.15252/embj.201695402 Medline

34. M. Kanke, E. Tahara, P. J. Huis In’t Veld, T. Nishiyama, Cohesin acetylation and Wapl-Pds5 oppositely regulate translocation of cohesin along DNA. *EMBO J.* **35**, 2686–2698 (2016). doi:10.15252/embj.201695756 Medline

35. G. Cameron, D. T. Gruszka, R. Gruar, S. Xie, Ç. Kaya, K. A. Nasmyth, J. Baxter, M. Srinivasan, H. Yardimci, Raw data for: Sister chromatid cohesion establishment during DNA replication termination, Figshare (2024); https://doi.org/10.25418/crick.25062113.

36. J. B. Grimm, T. A. Brown, B. P. English, T. Lionnet, L. D. Lavis, Synthesis of Janelia Fluor HaloTag and SNAP-tag ligands and their use in cellular imaging experiments. *Methods Mol. Biol.* **1663**, 179–188 (2017). doi:10.1007/978-1-4939-7265-4_15 Medline

37. E. Marklund, B. van Oosten, G. Mao, E. Amselem, K. Kipper, A. Sabantsev, A. Emmerich, D. Globisch, X. Zheng, L. C. Lehmann, O. G. Berg, M. Johansson, J. Elf, S. Deindl, DNA surface exploration and operator bypassing during target search. *Nature* **583**, 858–861 (2020). doi:10.1038/s41586-020-2413-7 Medline

38. R. Lebofsky, T. Takahashi, J. C. Walter, DNA replication in nucleus-free *Xenopus* egg extracts. *Methods Mol. Biol.* **521**, 229–252 (2009). doi:10.1007/978-1-60327-815-7_13 Medline

39. J. A. Wohlschlegel, B. T. Dwyer, S. K. Dhar, C. Cvetic, J. C. Walter, A. Dutta, Inhibition of eukaryotic DNA replication by geminin binding to Cdt1. *Science* **290**, 2309–2312 (2000). doi:10.1126/science.290.5500.2309 Medline

40. L. Loeff, J. W. J. Kerssemakers, C. Joo, C. Dekker, *AutoStepfinder*: A fast and automated step detection method for single-molecule analysis. *Patterns (N. Y.)* **2**, 100256 (2021). doi:10.1016/j.patter.2021.100256 Medline

41. P. Silver, Indirect immunofluorescence labeling in the yeast *Saccharomyces cerevisiae*. *Cold Spring Harb. Protoc.* **2009**, prot5317 (2009). doi:10.1101/pdb.prot5317 Medline

42. Y. Murayama, C. P. Samora, Y. Kurokawa, H. Iwasaki, F. Uhlmann, Establishment of DNA-DNA interactions by the cohesin ring. *Cell* **172**, 465–477.e15 (2018). doi:10.1016/j.cell.2017.12.021 Medline
