# Supplementary material for: Sister chromatid cohesion establishment during DNA replication termination
Source: Science. Author manuscript; Available in PMC 2024 Apr 5. (PMC7615807; doi:10.1126/science.adf0224)
Supplement: Supplementary Movies [file EMS194777-supplement-Supplementary_Movies.pdf]

Supplementary Movies

Movie S1. Sliding of JF646-cohesin (magenta) ahead of the replication fork. Nascent DNA labeled with Fen1-mKiKGR (red). Related to Fig. 1B.

Movie S2. LD555-labeled CMG pushing JF646-cohesin during fork progression. Related to Fig. 1D.

Movie S3. Cohesin relocalization to a DNA replication termination site. Related to Fig. 2A.

Movie S4. Visualization of CMG disassembly and cohesin retention at a replication termination site. Related to fig. S9B.

Movie S5. Collapse and separation of sister DNAs upon completion of DNA replication. Related to Fig. 3B.

Movie S6. JF549-cohesin associating with collapsed sister DNAs. Related to Fig. 3D.

Movie S7. Converging replisomes bypassing one another with one replisome continuing to push a labeled cohesin. Related to Fig. 4B.
